# Spatial Analysis of the Tumor Microenvironment in Diffuse Large B-cell Lymphoma Reveals Clinically Relevant Cell Interactions and Recurrent Cellular Neighborhoods

**DOI:** 10.1158/2326-6066.CIR-24-1163

**Published:** 2025-08-06

**Authors:** Matias Autio, Suvi-Katri Leivonen, Leo Meriranta, Marja-Liisa Karjalainen-Lindsberg, Teijo Pellinen, Sirpa Leppä

**Affiliations:** 1 Research Programs Unit, Applied Tumor Genomics, University of Helsinki, Helsinki, Finland.; 2 Department of Oncology, University of Helsinki and Helsinki University Hospital Comprehensive Cancer Center, Helsinki, Finland.; 3 iCAN Digital Precision Medicine Flagship, Helsinki, Finland.; 4 Department of Pathology, Helsinki University Hospital, Helsinki, Finland.; 5 Institute for Molecular Medicine Finland (FIMM), Helsinki, Finland.

## Abstract

Recent studies have explored the composition of the tumor microenvironment (TME) in diffuse large B-cell lymphoma (DLBCL) However, cell-to-cell interactions, along with the spatial organization of DLBCL TME and their impact on patient outcomes, have remained poorly characterized. We applied multiplex immunofluorescence, cell phenotyping, and neighborhood analysis to investigate 1,218,756 single cells in 99 samples from patients with primary DLBCL. We identified 17 cell phenotypes and 10 recurrent cellular neighborhoods (RCN) across samples, subdividing DLBCLs into immune-poor areas and areas with diverse immune cell infiltrates. Avoidance of B cells and PD-1^+^ T cells was associated with less aggressive clinical characteristics and favorable survival. Likewise, the proximity of CD8^+^ T cell–rich and immune-poor RCNs translated to favorable patient outcomes, and the proximity of PD-L1^+^ B cell–rich and CD8^+^ T cell–rich RCNs to unfavorable patient outcomes. Our findings provide insights into the spatial interactions and organization of DLBCL TME with implications for patient outcomes.

## Teaser

The authors identify clinically relevant cellular interactions and recurrent cellular neighborhoods in the TME of diffuse large B-cell lymphoma and characterize patient groups that could potentially benefit from TME-targeted therapies.

## Introduction

Diffuse large B-cell lymphoma, not otherwise specified (DLBCL NOS) is the most common aggressive lymphoid cancer in adults. Although anthracycline-based immunochemotherapy is curative in most patients (1), 30% to 40% of patients have primary refractory disease or experience progression later during follow-up, and despite the emergence of novel immunotherapies, such as chimeric antigen receptor (CAR) T cells or bispecific antibodies, about 50% of patients still die of lymphoma (2, 3). A major obstacle to improving patient outcome results from the inadequate disassembly of clinical and biological heterogeneity, and therefore novel tools that improve patient stratification are anticipated.

Based on the cell-of-origin (COO), DLBCL NOS is divided into germinal center B cell (GCB) and activated B cell–like (ABC) DLBCLs, and based on common genetic aberrations, genetic subtypes within the COO subtypes have been identified (4–7). More recently, novel DLBCL subtypes have also been proposed based on the composition of the tumor microenvironment (TME; refs. 8, 9). DLBCL TME can impact the clinical course of disease in diverse ways (10, 11). Whereas TME acts as a part of the body’s attempt to fight against lymphoma (10), it can, on the other hand, offer a growth advantage to lymphoma cells by secreting protumorigenic factors and protecting lymphoma cells from both internal and external threats (12). Different immune cells in the TME and their phenotypes have distinct effects on the clinical course of DLBCL, and regularities in the spatial organization of the TME have also been reported (13–21).

Lymphomas with different TMEs also have different molecular characteristics, highlighting the diverse mechanisms they recruit to survive in distinct environments. For instance, overactivation of the NF-κB pathway is common in inflamed lymphomas, and evasion from the rich immune infiltrate can occur by downregulation of HLA I and II or by upregulation of immune checkpoint molecules, such as PD-1 and PD-L1 (11, 22–28). However, unlike in other lymphomas, such as Hodgkin lymphoma, in which the blockade of these immune checkpoint molecules is an effective therapeutic approach, and despite the expression of PD-1 and PD-L1 in a significant proportion of DLBCLs, checkpoint blockade therapy has so far shown limited efficacy in this disease (29–31). Thus, predictive factors for the use of these therapies in DLBCL still need to be identified.

The cellular contexture of different cancers, including DLBCLs, has recently been found not to be arranged randomly but rather in an organized manner, in which cells form distinct cellular neighborhoods that are shared among individuals (19–21, 32–34). We previously showed that tumor-infiltrating immune cells divide DLBCLs into inflamed and noninflamed subgroups and that their proportions and immunophenotypes are associated with survival (18). However, spatially resolved information is required to further understand the immune pressures through cell-to-cell interactions that affect patient outcomes. In this study, we characterized immune cell composition, cell-to-cell interactions, and spatial architecture at the single-cell resolution in DLBCL samples from 99 treatment-naïve patients and correlated the findings with molecular, clinicopathologic, and survival data. We identified recurrent cellular neighborhoods (RCN), which are arranged similarly across different samples, and found that this spatial organization along with specific immune cell interactions has clinical significance.

## Materials and Methods

### Patients and samples

The multiplex immunofluorescence (mIF) cohort consisted of 99 patients diagnosed with primary DLBCL NOS between the years 2001 and 2013 (Table 1). Diagnostic tumor tissue was collected, fixed in formalin, and then embedded in paraffin for diagnostic staining and long-term storage. Patients were treated with rituximab, cyclophosphamide, doxorubicin, vincristine, and prednisone (R-CHOP) or R-CHOP–like immunochemotherapy. We excluded patients with high-grade B-cell lymphoma from the cohort. We constructed tissue microarrays (TMA) containing multiple (2, 3) 1-mm cores per patient from formalin-fixed paraffin-embedded primary diagnostic tumor tissue (35). RNA sequencing and exome sequencing data were previously produced for 61 cases (36). We determined lymphoma microenvironment (LME) subtypes and lymphoma ecotypes for the cases with RNA sequencing data (8, 9). Reanalyzed data on driver mutations were available for 85 patients, and LymphGen classifications were performed previously without considering copy-number alterations (bioRxiv 2023.11.21.567983; refs. 7, 37). The study was approved by the Ethics Committee of Helsinki University Hospital, Finland, the Finnish National Authority for Medicolegal Affairs (VALVIRA), and the Institutional Review Board. Sample collection was performed in accordance with the Declaration of Helsinki. Written informed consent was waived because of the retrospective nature of the study and the deidentification of the patient information. All methods were carried out following the World Health Organization and International Classification of Diseases-O-3 guidelines and recommendations.

**Table 1.** Patient demographics.

Characteristics	*n* (%)
Patients	99 (100)
Age	​
Median (range)	65 (18–84)
<60	40 (40)
≥60	59 (60)
Sex	​
Male	57 (58)
Female	42 (42)
COO	​
GCB	31 (31)
ABC	36 (36)
Unclassified	10 (10)
nd	22 (22)
Genetic subtype	​
EZB	8 (8)
MCD	6 (6)
BN2	12 (12)
ST2	6 (6)
N1	0 (0)
Other	53 (54)
nd	14 (14)
World Health Organization PS	​
0–1	72 (73)
≥2	26 (26)
nd	1 (1)
Stage	​
I–II	56 (57)
III–IV	42 (42)
nd	1 (1)
IPI	​
0–2	62 (63)
3–5	34 (34)
nd	3 (3)
Elevated LDH	​
Yes	52 (53)
No	45 (45)
nd	2 (2)
EN	​
0–1	81 (82)
≥2	15 (15)
nd	3 (3)
B symptoms	​
Yes	24 (24)
No	72 (73)
nd	3 (3)
Treatment	​
R-CHOP/R-CHOP-like	98 (99)
R-ICE	1 (1)
5-year PFS	72.1%
5-year OS	76.2%

Abbreviations: ABC, ABC-like; EN, extranodal site; GCB, GCB-like; LDH, lactate dehydrogenase; nd, not determined; PS, performance score; R-CHOP, rituximab, cyclophosphamide, doxorubicin, vincristine, and prednisone; R-ICE, rituximab, ifosfamide, carboplatin, and etoposide.

### mIF

We performed mIF on TMAs constructed from primary diagnostic formalin-fixed, paraffin-embedded tumor tissue using the protocol originally described by Blom and colleagues (38), with some modifications. A flowchart demonstrating the analysis pipeline is presented in Supplementary Fig. S1. We used a 12-plex panel to characterize B cells (CD20), T cells (CD3, CD4, CD8, Foxp3, and Tbet), macrophages (CD68 and CD163), and immune checkpoint molecules (PD-1, PD-L1, and CD96). The entire panel and the used antibodies are presented in Supplementary Table S1.

We first detected Tbet and CD96 using Alexa Fluor 488 and Alexa Fluor 555, which we amplified using tyramide signal amplification (PerkinElmer). We detected PD-L1 and FOXP3 using primary antibodies produced in different species and Alexa Fluor 647 and Alexa Fluor 750 fluorochrome-conjugated secondary antibodies and counterstained nuclei using 4',6-diamidino-2-phenylindole (DAPI). We then mounted the slides and acquired whole-slide fluorescence images. After performing the first round of staining, we removed the coverslips by washing the slides in wash buffer at 4°C. Then to remove the Alexa Fluor signals from the previous round of staining, we bleached the slides by first soaking the slides in TBS buffer containing 25 mmol/L NaOH and 4.5% H_2_O_2_. Finally, we denatured the antibodies from the previous round of staining by heating the slides in 1 mmol/L Tris/10 mmol/L EDTA, pH 9, solution for 20 minutes at 99°C. After washing the slides, we performed a second round of staining to detect CD4 and PD-1 by using Alexa Fluor 647 and Alexa Fluor 750 secondary antibodies. After acquiring whole-slide images, we again bleach-boiled the slides to remove the second-round staining and repeated the staining and washing steps to further perform three more rounds of staining to detect CD3, CD8, CD68, CD163, CD20, and CD45. Representative single-channel images displaying the staining of each antibody are shown in Supplementary Fig. S2.

Due to weak IF signal, Tbet was excluded from further analyses. We used Ilastik v.1.3.3 (RRID: SCR_015246) software to filter out areas with staining artifacts. Data on cases with an inflamed and noninflamed TME was available from previous mIF analyses (18). We also had available data on β_2_ microglobulin (B2M), HLA-ABC, and HLA-DR expression from previously performed IHC stainings (17).

### Imaging

We acquired all whole-slide fluorescence images at a resolution of 0.325 µm/pixel using Zeiss Axio Scan.Z1 with Zeiss 20X (0.8 NA, M27) Plan-Apochromat objective, Hamamatsu ORCA-Flash 4.0 V2 Digital CMOS camera (16-bit), and Zeiss Colibri.7 LED Light Source. For fluorescence imaging, we used DAPI cube (Zeiss Filter Set 02), FITC cube (Zeiss Filter Set 38 HE), Cy3 cube (Chroma Technology Corp 49004 ET CY3/R), Cy5 cube (Chroma Technology Corp 49006 ET CY5), and Cy7 cube (Chroma Technology Corp 49007 ET CY7) filter sets. After image acquisition, we converted the images to JPEG2000 format at 100% quality and stored them as 8-bit greyscale images.

### Cell segmentation

We segmented nuclei using NucleAIzer (RRID: SCR_026500), a pretrained deep learning segmentation model (39). We then used CellProfiler v.3.1.8 software (RRID: SCR_007358) to expand nuclei masks by five pixels to receive final cell masks (Supplementary Fig. S3). Objects with an area <100 pixels were removed from further analyses. We quantified marker intensities for each cell using histoCAT software (RRID: SCR_026499; ref. 40).

### Cell phenotyping

For cell phenotyping, we first performed the Phenograph algorithm (100 neighbors) using the Rphenograph (RRID: SCR_022603) package in R v.4.3.1 (RRID: SCR_001905) to receive 33 different clusters (41). Based on marker expression and using Napari image viewer (RRID: SCR_022765) to overlap the Phenograph clusters on the original images, we then grouped the 33 Phenograph clusters into eight main cell types, namely B cells, CD4^+^ T cells, CD8^+^ T cells, regulatory T cells (Treg), CD163^−^ macrophages, CD163^+^ macrophages, other immune cells, and nonimmune cells. For simplicity, in this article, we use the terms M1-like macrophages for CD163^−^ or antitumorigenic macrophages and M2-like macrophages for CD163^+^ or protumorigenic macrophages. Unspecific Phenograph clusters containing cells from more than one main cell type were divided by manual thresholding of CD4, CD8, CD20, and CD163 using the sm.pl.gate_finder function in the scimap package (RRID: SCR_024751; Python v3.9, RRID: SCR_008394; ref. 32). Finally, we manually set the threshold for positivity for PD-1, PD-L1, and CD96 to receive a final set of 17 cell types. We confirmed that the cell annotations were correct in Napari image viewer by overlapping the original images with the final cell phenotypes. Due to leakage of signal from adjacent cells, many non-B cells falsely seemed to be positive for CD20. For visualizing of how the identified cell types clustered in relation to each other, we produced a t-distributed stochastic neighbor embedding plot using the Rtsne package (RRID: SCR_016342) in R. We selected a perplexity value of 30 to balance the local and global structure based on the dataset size (total number of cells = 60,938).

### Cell interaction analyses

We performed cell–cell interaction analyses using the scimap package (Python V3.9; ref. 32). We used the sm.tl.spatial_interaction function to calculate the probability of cell types interacting with each other, compared with a random background (1,000 permutations) in each sample. We used a radius of 60 pixels (20 µm) to define interacting cells. We used the sm.tl.spatial_distance function to calculate the average shortest distance between different cell types and cells with different cellular neighborhoods.

### Identification of RCNs

We identified RCNs by first constructing a neighborhood matrix using the defined cell phenotypes, followed by latent Dirichlet allocation with 10 motifs (default) using the sm.tl.spatial_lda function in the scimap package (Python v3.9; refs. 32, 42). We defined a cellular neighborhood to consist of 20 cells (self and 19 nearest neighbors; refs. 20, 21). Twenty neighbors were chosen to define a neighborhood because it provided the best balance between capturing local interactions and minimizing noise (Supplementary Fig. S4). We then used K-means clustering with 10 clusters to identify RCNs. We defined the optimal number of clusters by looking for the elbow point in a computed scree plot.

### Statistical analyses

We performed all statistical analyses with R v.4.3.1 and Python v3.9. For unsupervised hierarchical clustering analyses, we used Euclidean distance and Ward linkage in the pheatmap package. We estimated the prognostic significance of each variable using univariable and multivariable Cox proportional hazards regression models and the difference in survival between different patient groups using the Kaplan–Meier method with the log-rank test. Overall survival (OS) and progression free survival (PFS) were defined as the time from diagnosis to death from any cause and the time from diagnosis to progression or death from any cause, respectively. We sought the optimal cutoff for the distance between different RCNs using the maxstat R package (RRID: SCR_025679). We compared two or more groups using Mann–Whitney U and Kruskal–Wallis H tests, respectively. We corrected *P* values for errors caused by multiple testing using the Benjamini–Hochberg method. Adjusted *P* values <0.05 were considered significant.

### Data availability

The mIF images, as well as the raw single-cell data extracted from the images, have been uploaded to Zenodo, https://zenodo.org/, with access number 15362450 (DOI: 10.5281/zenodo.15362450). All other data are available in the article and its supplementary files or from the corresponding author upon reasonable request.

## Results

### The cellular composition of the TME in DLBCL NOS

The study population consisted of 99 patients with primary DLBCL NOS treated with standard immunochemotherapy. Patient demographics are described in Table 1. During the median follow-up time of 55 months (IQR: 43 months; 70 months), 19 patients experienced a relapse and 21 patients died, translating to a 5-year PFS of 71% and a 5-year OS of 75%.

**Figure 1.**
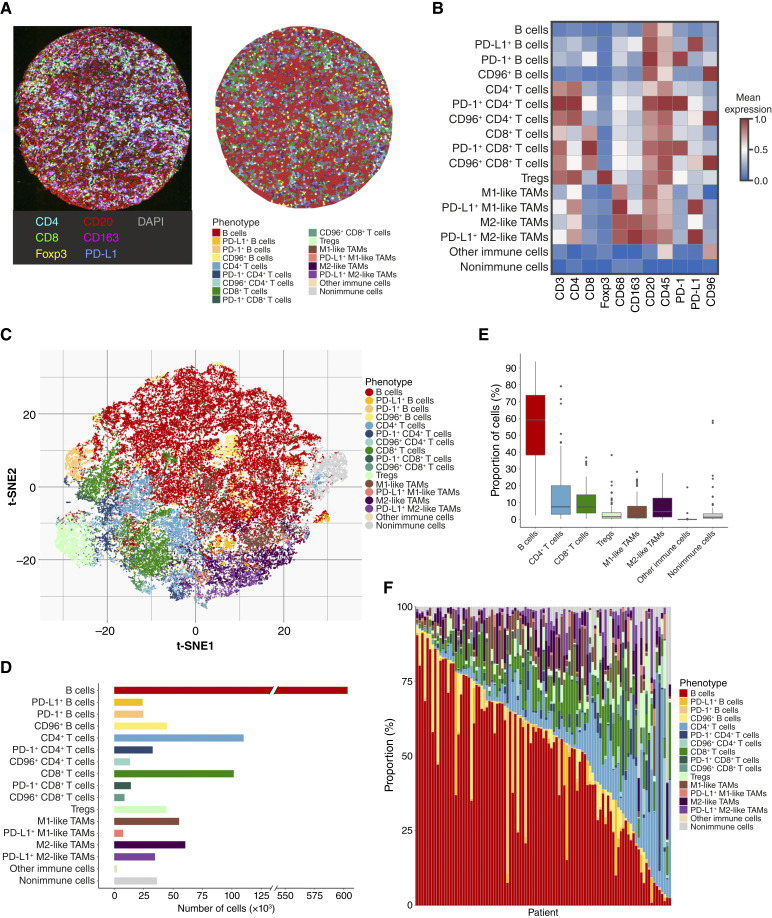
Cellular composition of DLBCL NOS (*n* = 99) characterized using mIF. Cells were phenotyped using the Phenograph algorithm followed by manual thresholding of particular markers to divide unspecific Phenograph clusters. **A,** Representative image of a TMA core stained with mIF and a Voronoi plot of the same core with cell phenotypes labeled. **B,** A heatmap with mean expression values of the stained markers in each identified cell phenotype. **C,** A t-distributed stochastic neighbor embedding (t-SNE) plot performed with the expression of all stained markers in the segmented cells; 5% (60,938 cells) of the cells were randomly selected for plotting. **D,** A barplot depicting the total number of identified cells of each cell phenotype. **E,** A boxplot depicting the median proportions of different cell phenotypes in the DLBCL NOS samples. Lower and upper hinges of the boxplot correspond to the 25^th^ and 75^th^ percentiles, and whiskers the confidence intervals 1.5 × IQR from the hinge. **F,** A barplot depicting the cellular composition of the lymphomas in each patient in the cohort (*n* = 99).

First, we characterized the composition of the TME in DLBCL NOS. We identified 1,218,756 single cells with a median of 12,354 (range, 2,504–28,671) cells per sample. Based on phenotyping using the Phenograph algorithm and manual thresholding, we identified 17 different cell phenotypes, covering distinct B cells, T cells, and tumor-associated macrophages (TAM; Fig. 1A–D). We also identified a group with other immune cells and a nonimmune cell group. The mean expression of the used markers in the identified cell phenotypes is presented in Fig. 1B. Cellular composition of the samples varied greatly (Fig. 1E and F). As expected, B cells were the most common cell type with an average proportion of 55% (range, 2.4%–94%). Within the TME, CD4^+^ Th cells represented the most common cell type (mean, 14%, range, 0.17%–79%), followed by CD8^+^ cytotoxic T cells (mean, 9.9%, range, 0.30%–37%) and M2-like TAMs (mean, 7.9%, range, 0%–28%). M1-like TAMs accounted on average for 5.4% (range, 0.06%–28%) of the cells and Tregs for 3.6% (range, 0%–38%), whereas other immune cell types accounted for 0.26% (range, 0%–19%) and nonimmune cells for 4.0% (range, 0.05%–59%) of the total number of cells in the samples. Of all CD4^+^ Th cells, 20.9% expressed PD-1, whereas PD-1 expression was present in 11.4% of the CD8^+^ T cells. PD-L1 expression was characteristic of M2-like TAMs, of which 36.5% expressed PD-L1, whereas only 12.2% of M1-like TAMs and 4.01% of B cells expressed PD-L1. M2-like TAMs seemed to be more common in the TME of ABC DLBCLs, whereas the proportion of M1-like TAMs and Tregs was higher in GCB DLBCLs (adj. *P* = 0.058 for all; Supplementary Fig. S5). There was no correlation between the expression of HLA-ABC, B2M, or HLA-DR and the proportion of any immune cell types (Supplementary Fig. S6). Neither were the proportions of cell phenotypes associated with the patient outcomes (Supplementary Fig. S7).

### Avoidance of B cells and PD-1^+^ T cells is associated with less aggressive disease and favorable survival

**Figure 2.**
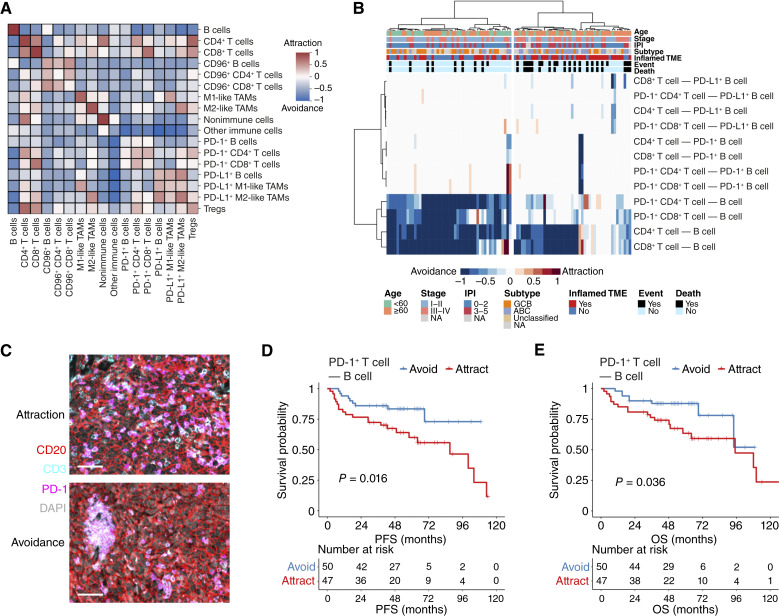
Cellular interactions in DLBCL NOS estimated using a permutation test (1,000 permutations and using a radius of 20 µm) to define interacting cells. **A,** A heatmap depicting the attraction and avoidance of identified cell types in DLBCL NOS. The studied cell phenotypes are depicted on the *y*-axis, and the neighboring cell phenotypes on the *x*-axis. Red color means that the neighboring cell type is attracted to the studied cell type, and blue color that the neighboring cell type is avoiding the studied cell type. **B,** Unsupervised hierarchical clustering (Euclidean distance and Ward linkage) of different T cell–B cell interactions in lymphoma samples in patients with DLBCL NOS (*n* = 98). Interactions are depicted on the *y*-axis and patients on the *x*-axis. Red color indicates attraction and blue color indicates avoidance between the studied cell types. CD4^+^ T cell, CD8^+^ T cell, and B cell indicate cells not expressing PD-1 or PD-L1. One sample lacked interactions between T cells and B cells and was excluded from the analysis. **C,** Representative images from mIF-stained TMA cores in which B cells are attracted to as well as avoiding PD-1^+^ T cells, respectively. Scale bar, 50 µm. **D** and **E,** Kaplan–Meier (log-rank test) survival plots depict PFS (**D**) and OS (**E**) in R-CHOP–treated patients with DLBCL in whose lymphomas B cells are avoiding and attracted to PD-1^+^ T cells based on the clustering in (**B**). NA, not applicable; R-CHOP, rituximab, cyclophosphamide, doxorubicin, vincristine, and prednisone.

Next, we sought to explore the spatial interactions of immune cells within DLBCL samples. First, we studied which cell types were attracted to each other and which cell types avoided each other (Fig. 2A). We found that many immune cells primarily interacted with cells having the same phenotype, suggesting that similar immune cell types are often localized in each other’s vicinity (Fig. 2A). In contrast, B cells tended to avoid especially different T cells and PD-L1^+^ M2-like TAMs.

To study whether cell interactions varied across samples, we performed unsupervised hierarchical clustering with the interactions between all identified cell types (Supplementary Fig. S8). We found great heterogeneity regarding which cell types were attracted and which cell types avoided each other across the samples. Especially interactions between immune cells and B cells and interactions between different T cells varied. To find out whether certain cell interaction patterns were associated with clinical characteristics and patient outcomes, we performed unsupervised hierarchical clustering on different T cell–B cell interactions. DLBCLs clustered into two groups based on the interactions between B cells and PD-1^+^ T cells (Fig. 2B and C). The first group consisted of cases in which B cells avoided interactions with PD-1^+^ T cells. Conversely, the second group consisted of cases in which B cells were more attracted to PD-1^+^ T cells while avoiding interaction with PD-1^−^ T cells. Lymphomas in which B cells avoided PD-1^+^ T cells had a higher proportion of B cells, as well as PD-1^+^ CD4^+^ T cells and PD-1^+^ CD8^+^ T cells (adj. *P* = 0.006, adj. *P* < 0.001, and adj. *P* < 0.001, respectively; Supplementary Fig. S9). Correlation of these groups with clinical factors revealed that when B cells avoided PD-1^+^ T cells, but to a lesser extent also PD-1^−^ T cells, lymphomas represented more often GCB than ABC phenotype (41% vs. 22%, *P* = 0.023) and the patients had lower International Prognostic Index (IPI) scores (IPI 0–2: 76% vs. 49%, *P* = 0.020; Supplementary Table S2). PD-1^+^ T-cell avoidance and favorable clinical features also translated to favorable survival (5-year PFS: 84.9% vs. 60.3%, *P* = 0.011; 5-year OS: 89.8% vs. 63.7%, *P* = 0.025; Fig. 2D and E).

### Identification of RCNs in DLBCL NOS

**Figure 3.**
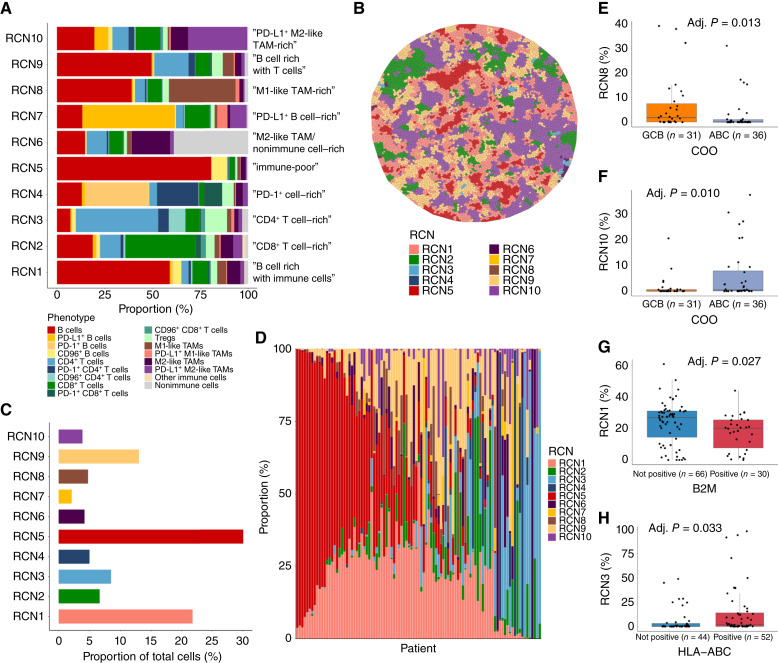
Identification of RCNs in DLBCL NOS (*n* = 99) by creating a neighborhood matrix followed by a latent Dirichlet allocation analysis and K-means clustering. **A,** A barplot depicting the average cellular composition of each of the identified RCNs. **B,** A Voronoi plot of the same TMA core as presented in Fig. 1A with cells colored based on their RCNs. **C,** A barplot depicting the proportion of all cells that present with each of the identified RCNs. **D,** A barplot depicting the RCN composition of the lymphomas in each patient in the cohort. **E** and **F,** Boxplots depicting the median proportion of cells with an M1-like TAM-rich RCN8 (**E**) and PD-L1^+^ M2-like TAM-rich RCN10 (**F**) in GCB and ABC DLBCLs. **G** and **H,** Boxplots depicting the median proportion of cells with a B cell rich with immune cells RCN1 in B2M positive and negative DLBCLs (**G**) and the proportion of cells with a CD4^+^ T cell–rich RCN3 in HLA-ABC–positive and –negative DLBCLs (**H**). The groups were compared using Mann–Whitney U test. Lower and upper hinges of the boxplot correspond to the 25th and 75th percentiles, and whiskers the confidence intervals 1.5 × IQR from the hinge.

To further uncover the spatial structures in the TME of DLBCL NOS, we investigated whether cells are regularly organized in cellular neighborhoods that are shared between samples. To identify RCNs, we first created a neighborhood matrix of all cells and cellular phenotypes with their 19 nearest neighbors and performed a latent Dirichlet allocation analysis (42). We then performed K-means clustering and identified 10 RCNs with distinct cell compositions (Fig. 3A and B; Supplementary Fig. S10). The most common RCN was an immune-poor neighborhood RCN5 (30.1% of cells), followed by neighborhoods RCN1 (B-cell rich with immune cells, 21.7%) and RCN9 (B-cell rich with T cells, 13.0%). The proportions of all RCNs are presented in Fig. 3C. DLBCL samples varied greatly in their RCN compositions (Fig. 3D). However, each RCN was present in at least 39% (39/99) of the samples, with PD-1^+^ cell–rich RCN4 being present in the smallest number of samples.

GCB and ABC DLBCLs also differed in their RCN composition. Specifically, GCB DLBCLs consisted of a larger proportion of the M1-like TAM-rich RCN8 (adj. *P* = 0.013), whereas the PD-L1^+^ M2-like TAM-rich RCN10 was more abundant in ABC DLBCLs (adj. *P* = 0.010; Fig. 3E and F). Furthermore, the B cell rich with immune cells RCN1 was more common in GCB DLBCLs (Supplementary Fig. S11).

When we correlated the RCN compositions in DLBCLs with the expression of HLA and B2M, we found that B2M and HLA-ABC–positive lymphomas consisted of a smaller proportion of the B cell rich with immune cells RCN1 (adj. *P* = 0.027 and adj. *P* = 0.033; Fig. 3G; Supplementary Fig. S12A–S12C). Conversely, HLA-ABC–positive lymphomas consisted of a larger proportion of the CD4^+^ T cell–rich RCN3 and PD-1^+^ cell–rich RCN4 (adj. *P* = 0.033 and adj. *P* = 0.002; Fig. 3H; Supplementary Fig. S12B and S12D). Lastly, HLA-DR–positive lymphomas consisted less of the M2-like TAM-rich/nonimmune cell–rich RCN6 and more of the B cell rich with T cells RCN9 (adj. *P* = 0.033 and adj. *P* = 0.033; Supplementary Fig. S12E–S12G).

### The spatial organization of RCNs in DLBCL NOS

**Figure 4.**
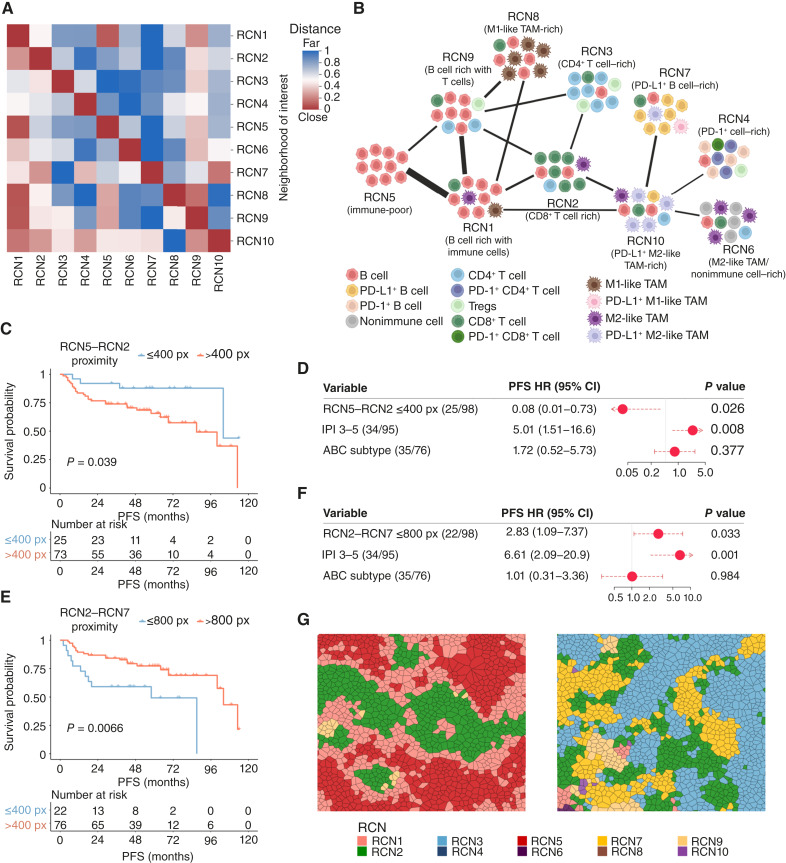
The organization of RCNs in DLBCL NOS calculated using the average distance between the cells in different RCNs. **A,** A heatmap depicting the average distances from cells in the RCNs shown on the *y*-axis (neighborhood of interest) to the cells in the RCNs depicted on the *x*-axis. The distance from neighborhood *x* to neighborhood* y* represents the average distance of a cell in neighborhood *x* to the nearest cell in neighborhood *y*, whereas the distance from neighborhood *y* to neighborhood *x* signifies the average distance of a cell in neighborhood *y* to the nearest cell in neighborhood *x*. **B,** Graphical illustration of the organization of RCNs in DLBCL NOS. The RCN that is, on average, situated closest to another RCN is connected to that RCN by a line. RCNs not connected by a line are, on average, situated further apart from each other. The distances between cells in neighborhoods *x* and *y* are calculated as the average of the distance from cells in neighborhood *x* [the neighborhood of interest in (**A**)] to cells in neighborhood *y*, and the distance from cells in neighborhood *y* to cells in neighborhood *x*. The thickness of the line is inversely proportional to the distance between cells in the neighborhoods. **C,** Kaplan–Meier (log-rank test) survival plot depicts PFS in rituximab, cyclophosphamide, doxorubicin, vincristine, and prednisone (R-CHOP)–treated patients with DLBCL NOS in whose lymphomas CD8^+^ T cell–rich RCN2 neighborhoods are situated close to immune-poor RCN5 neighborhoods, and patients in whose lymphomas these RCNs are situated far from each other or are not present. The optimal cutoff was sought using the R maxstat package. **D,** A forest plot visualizing the impact of the proximity of CD8^+^ T cell–rich RCN2 neighborhoods to immune-poor RCN5 neighborhoods on PFS in a Cox multivariable regression analysis with IPI and COO in R-CHOP–treated patients with DLBCL NOS. **E,** Kaplan–Meier (log-rank test) survival plot depicts PFS in R-CHOP–treated patients with DLBCL NOS in whose lymphomas PD-L1^+^ B cell–rich RCN7 neighborhoods are situated close to CD8^+^ T cell–rich RCN2 neighborhoods, and patients in whose lymphomas these RCNs are situated far from each other or are not present. The optimal cutoff was sought using the R maxstat package. **F,** A forest plot visualizing the impact of the proximity of PD-L1^+^ B cell–rich RCN7 neighborhoods to CD8^+^ T cell–rich RCN2 neighborhoods on PFS in a Cox multivariable regression analysis with IPI and COO in R-CHOP–treated patients with DLBCL NOS. **G,** Voronoi plots of representative samples in which CD8^+^ T cell–rich RCN2 neighborhoods are situated close to immune-poor RCN5 neighborhoods (left) and PD-L1^+^ B cell–rich RCN7 neighborhoods are situated close to CD8^+^ T cell–rich RCN2 neighborhoods (right). px, pixel. [**B,** Created in BioRender. Autio, M. (2025) https://BioRender.com/dnqcyry]

To identify the spatial organization of different neighborhoods, we studied the average distances between the cells in different neighborhoods (Fig. 4A and B; Supplementary Table S3). First, we found that immune-poor RCN5 neighborhoods were located close to RCN1 and RCN9, both of which are B cell–rich but also have immune cell infiltrates. Cells in the RCN1 and RCN9 neighborhoods were situated close to each other, as well as in the proximity of CD8^+^ T cell–rich RCN2 and M1-like TAM-rich RCN8. In addition, cells in the RCN9 were also located close to cells in the CD4^+^ T cell–rich RCN3. Cells in the RCN2 were also close to cells in the PD-L1^+^ B cell–rich RCN7 and PD-L1^+^ M2-like TAM-rich RCN10. Lastly, cells in the PD-1^+^ cell–rich RCN4, M2-like TAM/nonimmune cell–rich RCN6, and PD-L1^+^ B cell–rich RCN7 were located close to cells in the PD-L1^+^ M2-like TAM-rich RCN10. To conclude, we could identify immune-poor cell compartments in DLBCL NOS, which gradually shift to immune-rich cell compartments. Moreover, PD-1^+^ and PD-L1^+^ cell compartments are closely related to each other and separated from the immune-poor cell compartments.

### The organization of RCNs in DLBCL NOS is associated with survival

To study whether the organization of RCNs is associated with patient outcomes, we analyzed the survival of patients in whose lymphomas certain RCNs were located close to each other as compared with the patients in whose lymphomas these RCNs were located far away from each other or were not present. Survival was better in patients in whose lymphomas CD8^+^ T cell–rich RCN2 neighborhoods were situated less than 400 pixels (133 µm) away from the immune-poor RCN5 neighborhoods (5-year PFS: 87.8% vs. 65.7%, *P* = 0.039; Fig. 4C and G; Supplementary Fig. S13A). The impact of the adjacent location of these neighborhoods was independent of the IPI and COO (Fig. 4D). Conversely, the outcome was worse in patients in whose lymphomas PD-L1^+^ B cell–rich RCN7 neighborhoods were located less than 800 pixels (267 µm) away from CD8^+^ T cell–rich RCN2 neighborhoods, independent of the IPI and COO (5-year PFS: 49.2% vs. 77.4%, *P* = 0.007; Fig. 4E–G; Supplementary Fig. S13B and S13C). Furthermore, OS was also inferior when PD-L1^+^ M2-like TAM-rich RCN10 neighborhoods were closer than 400 pixels from CD8^+^ T cell–rich RCN2 neighborhoods (5-year OS: 60.7%% vs. 79.6%, *P* = 0.013; Supplementary Fig. S13D–S13F). The proportion of CD8^+^ T cell–rich RCN2 and immune-poor RCN5 was higher in lymphomas in which these RCNs were situated close to each other (adj. *P* < 0.001 and adj. *P* = 0.027), and the proportion of CD8^+^ T cell–rich RCN2 and PD-L1^+^ B cell–rich RCN7 was higher in lymphomas in which these RCNs were situated close to each other (adj. *P* = 0.034 and adj. *P* < 0.001; Supplementary Fig. S14). However, the proportion of these RCNs alone was not associated with outcome, indicating that the organization of these RCNs in relation to each other is clinically important.

### RCNs in different LME subtypes, lymphoma ecotypes, and genetic subtypes

**Figure 5.**
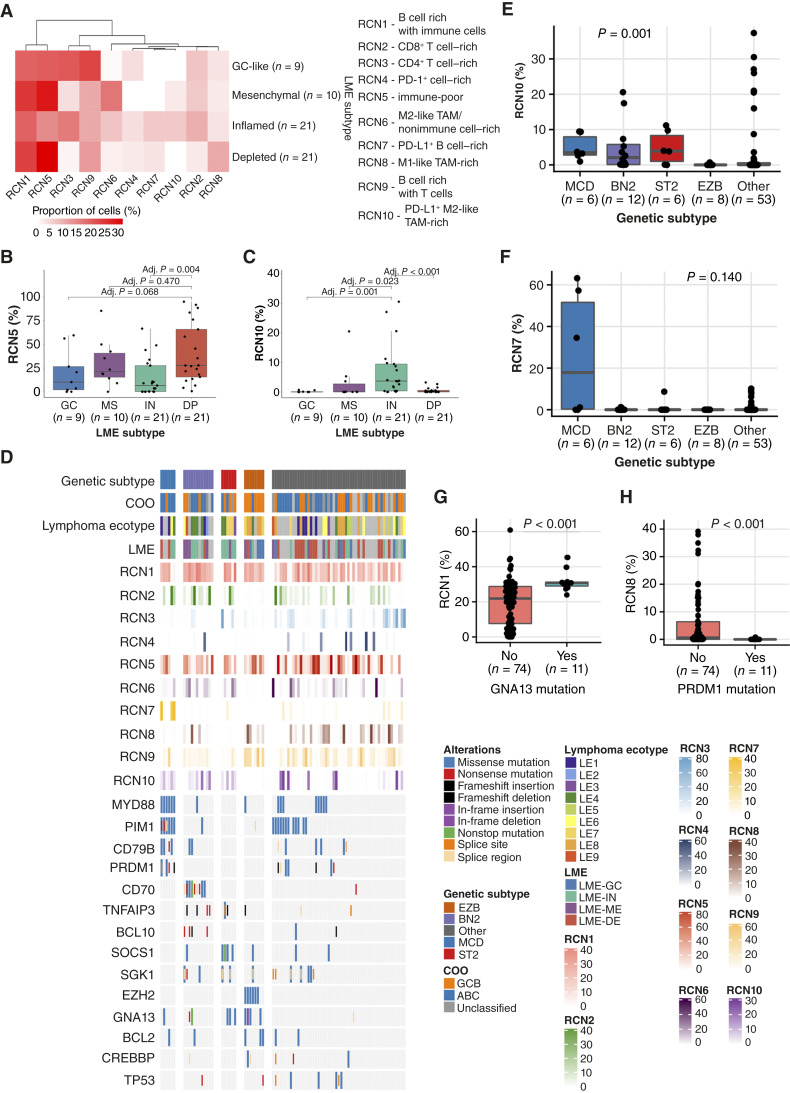
The relation between RCNs and LME subtypes and DLBCL genetic subtypes based on LymphGen classification and driver mutations (*n* = 85). **A,** A heatmap depicting the average proportions of RCNs in the different LME subtypes. **B,** A boxplot depicting the median proportion of cells with an immune-poor RCN5 neighborhood in depleted (DP) DLBCLs compared with GC-like (GC), mesenchymal (MS), and inflamed (IN) DLBCLs. The groups were compared using Mann–Whitney U test. **C,** A boxplot depicting the median proportion of cells with a PD-L1^+^ M2-like TAM-rich RCN10 neighborhood in inflamed DLBCLs compared with GC-like, mesenchymal, and depleted DLBCLs. The groups were compared using Mann–Whitney U test. **D,** Oncoprint of selected mutations and proportions of RCNs in DLBCL genetic subtypes. **E,** A boxplot depicting the median proportion of cells with a PD-L1^+^ M2-like TAM-rich RCN10 neighborhood in different genetic subtypes. The groups were compared using Kruskal–Wallis H test. **F,** A boxplot depicting the median proportion of cells with a PD-L1^+^ B cell–rich RCN7 neighborhood in different genetic subtypes. The groups were compared using Kruskal–Wallis H test. **G,** A boxplot depicting the median proportion of cells with a B cell–rich and immune cell–rich RCN1 neighborhood in DLBCLs with and without *GNA13* mutations. The groups were compared using Mann–Whitney U test. **H,** A boxplot depicting the median proportion of cells with a M1-like TAM-rich RCN8 neighborhood in DLBCLs with and without *PRDM1* mutations. The groups were compared using Mann–Whitney U test. Lower and upper hinges of the boxplots correspond to the 25th and 75th percentiles, and whiskers to the confidence intervals 1.5 × IQR from the hinge.

We next sought to identify whether the RCNs correlated with the subtypes determined by the composition of TME (8, 9). As expected, we found immune-poor RCN5 neighborhoods to be especially common in the depleted LME subtype, whereas the M2-like TAM/nonimmune cell–rich RCN6 neighborhoods were especially common in the mesenchymal LME subtype (Fig. 5A and B). The inflamed LME subtype was characterized by having higher proportions of the PD-L1^+^ B cell–rich and M2-like TAM–rich RCN7 and RCN10 neighborhoods compared with the others (Fig. 5A and C).

Our sample size limited the study of the RCN composition in different lymphoma ecotypes (9). However, we found that T cell–rich RCN2 and RCN3 as well as the PD-1^+^ cell–rich RCN4 and M1-like TAM-rich RCN8 neighborhoods were common in DLBCLs with an LE5 ecotype (Supplementary Fig. S15). Immune-poor RCN5 neighborhoods were especially found in DLBCLs with an LE8 ecotype, whereas M2-like TAM/nonimmune cell–rich RCN6 neighborhoods were found in DLBCLs with an LE9 ecotype and B cell rich with immune cells RCN1 neighborhoods in DLBCLs with a LE1 or LE3 ecotype (Supplementary Fig. S15).

Finally, we correlated the presence of certain RCNs with common driver mutations and genetic subtypes (Fig. 5D; Supplementary Table S4). Compared with other genetic subtypes, PD-L1^+^ M2-like TAM-rich RCN10 was rare in the EZB type (*P* = 0.001), which is in line with RCN10 being uncommon in GCB DLBCLs (Fig. 5E). Additionally, cases consisting of very high proportions of PD-L1^+^ B cell–rich RCN7 were MCD type, although when comparing median proportions, statistical significance was not reached (*P* = 0.140; Fig. 5F). Individually, the presence of *GNA13* mutations was associated with a high proportion of B cell rich with immune cells RCN1 neighborhoods (*P* < 0.001), whereas the presence of *PRDM1* mutations was associated with a lack of M1-like TAM-rich RCN8 neighborhoods (*P* < 0.001; Fig. 5G and H).

## Discussion

The TME of DLBCL has been previously dissected to determine subtypes based on the TME composition and identify clinically relevant immune cell subtypes (8, 9, 13–18). However, cellular interactions and spatial architecture have remained less studied. In this study, we explored the landscape of cellular interactions in DLBCL TME. We found that immune cells tend to be close to cells of the same phenotype and B cells avoid interaction with especially different T cells and M2-like TAMs. We also found that avoidance of B cells and PD-1^+^ T cells correlate with a less aggressive clinical course of the disease and favorable survival. Furthermore, we identified 10 RCNs, characterized by different immune cell infiltration patterns across DLBCL cases. The organization of these RCNs was also associated with patient outcomes, with the proximity of CD8^+^ T cell–rich RCNs to immune-poor RCNs being associated with better survival and the proximity PD-L1^+^ B cell–rich RCNs to CD8^+^ T cell–rich RCNs being associated with worse survival. Finally, we linked these RCNs with different microenvironmental and genomic DLBCL subtypes. In summary, our RCN analysis provides spatial and contextual insights, whereas previous studies have often relied on bulk profiling or isolated marker expression without considering the spatial organization of distinct TME cells.

We have previously shown that immune checkpoint–expressing TAMs are associated with unfavorable outcomes in inflamed DLBCLs and that TAMs interacting with T cells are more likely to express PD-L1 than TAMs not interacting with T cells (18). We speculated that TAMs are inhibiting the antitumoral activity of these T cells. In this study, we found that the attraction of B cells with PD-1^+^ T cells is associated with unfavorable survival. In DLBCLs in which B cells were attracted to PD-1^+^ T cells, the B cells were, conversely, avoiding PD-1^−^ T cells. Our results further imply that the advantageous role of T cells in fighting lymphoma is constrained by mechanisms involving immune checkpoint molecules. Nevertheless, functional studies showing that TAMs and B cells can inhibit T-cell activity by mechanisms involving immune checkpoint molecules in DLBCL are warranted.

Recently, consistent cellular neighborhoods in DLBCLs have been disclosed (19–21, 43). Colombo and colleagues analyzed 33 DLBCL cases and identified nine neighborhoods, which were based on tumor cell distance to the TME. They further grouped these clusters into three zones and identified immune desert regions that were surrounded by dispersed regions followed by immune-rich regions. These findings are in line with our results on immune-poor neighborhoods, which gradually shifted toward immune cell–rich neighborhoods. In another study, Reiss and colleagues (21) identified eight cellular neighborhood clusters in 30 and 55 DLBCL cases, which included high-grade BCLs. These neighborhoods included tumor-rich, CD4-rich, CD8-rich, and TAM-rich neighborhoods corresponding to similar RCNs as those in our study. In contrast to Reiss and colleagues, we utilized the expression of immune checkpoint molecules on immune cells in our neighborhood analysis and identified 10 RCNs, including separate PD-1^+^ cell–rich, PD-L1^+^ B cell–rich, and PD-L1^+^ M2-like TAM-rich RCNs. We also studied the spatial organization of the RCNs. Our findings strengthen the hypothesis that functional T cells that get close to the malignant cell compartments are important in the beneficial immune response against lymphoma.

We also correlated the RCNs with previously identified TME-specific DLBCL subtypes and ecotypes (8, 9). Immune-poor neighborhoods were common in DLBCLs classified as immune depleted, whereas PD-L1^+^ B cell–rich and M2-like TAM-rich neighborhoods were found especially in the inflamed DLBCLs. We also found a connection between RCNs and lymphoma ecotypes, although our analysis was limited by the small sample size.

Genomic profiling has refined the taxonomy of DLBCL (6, 7); yet, the connection between common mutations and TME in DLBCL has, to date, remained poorly characterized. Inactivating mutations in *B2M* and *HLA-I* and downregulation of MHC class I have been associated with reduced immunosurveillance (44, 45). We found an association between *GNA13* mutations and a higher proportion of B cell/immune cell–rich RCNs. GNA13 is regarded as a tumor suppressor in DLBCL (46), and altered GNA13 signaling could enhance the proliferation and survival of B cells in the TME, leading to B cell–rich neighborhoods. *PRDM1* (BLIMP-1) mutations were associated with a lack of M1-like TAM-rich RCNs. Loss of PRDM1/BLIMP-1 function has been shown to contribute to poor prognosis in ABC-like DLBCL (47). The absence of M1-like TAMs could suggest a shift toward an immunosuppressive TME, which could promote tumor progression by protecting the tumor from immune-mediated destruction and supporting tumor cell survival and proliferation.

PD-L1^+^ M2-like TAM-rich RCNs were absent from EZB-type DLBCLs, whereas PD-L1^+^ B cell–rich RCNs were identified in selected MCD-type DLBCLs. Altogether, these findings indicate that the microanatomic composition of the TME differs between molecularly defined DLBCL subtypes, which could translate to the differential efficacy of immuno-oncologic therapies that should be explored in future DLBCL trials.

The findings reveal potential therapeutic vulnerabilities and challenges. Despite the poor efficacy of PD-1– and PD-L1–targeting immunotherapies in DLBCL (30), our data imply that PD-1 expression on T cells regarding their interaction with B cells is clinically important. Therefore, it is tempting to speculate that PD-1 blockade has efficacy in patients whose T-cell activity is inhibited by the activation of PD-1 because of interactions with B cells, further highlighting the importance of careful patient selection. Furthermore, our discovery of RCNs, and the association of their spatial organization with patient outcomes, raises the intriguing possibility for clinical implications. In general, spatial insights provided by RCN analysis could have direct implications for the development of T cell–directed therapies, such as checkpoint inhibitors and CAR T-cell therapy, because the efficacy of these treatments may depend on preexisting spatial relationships within the TME. In addition, considering for example that the proximity of CD8^+^ T cell–rich and immune-poor RCNs are associated with favorable survival, we anticipate that certain immunologic therapies, such as bispecific antibodies, show better efficacy in those patients in whose lymphomas T-cell and tumor cell compartments are located close to each other. Collectively, the results underscore the importance of investigating the predictive value of RCNs in the context of immunotherapies in DLBCL.

Our results are consistent with the principal notion of immune-poor and immune cell–rich cellular neighborhoods (19, 21). Despite our study being limited to a 12-marker antibody panel, we identified 17 main immune cell subtypes in DLBCL TME, including immune checkpoint–expressing cells. For simplicity, in this work, we used the term M1-like TAMs to represent antitumorigenic macrophages and M2-like TAMs to represent protumorigenic macrophages. Although widely used, this dichotomy can today be considered oversimplified, because emerging evidence highlights a far more complex and dynamic spectrum of macrophage phenotypes. Therefore, a more detailed analysis of the macrophages that make up M1- and M2-like TAM-rich neighborhoods is warranted to confirm the function of these macrophages. Additionally, our cohort is considerably larger than the ones reported previously, allowing us to correlate our findings with clinical characteristics and survival. This highlights the robustness of a lower-plex antibody panel to identify the most evident cellular neighborhoods in the TME of DLBCL. In this study, our primary aim was to characterize the most prominent immune cell phenotypes in the TME of DLBCL and identify RCNs based on the organization of these cell types using a set of well-established markers. However, validation of the robustness of these RCNs using a higher-plex panel or spatial transcriptomics is warranted. It will also be important to validate our findings in additional datasets and to determine whether the RCNs and clinical associations we describe for primary DLBCLs persist over time and at relapse.

To conclude, we found translationally relevant interactions in the TME of DLBCL NOS that indicate a dependency on immune checkpoint molecules in the inhibition of the immune response against the lymphoma. Additionally, we found that DLBCL NOS is composed of 10 RCNs that vary in their frequency and spatial organization, which has clinical significance. The knowledge gained from this study increases our understanding of the spatial organization of DLBCL and may have translational potential in the selection of patients for immunotherapies.

## Supplementary Material

Tables S1-S3 Tables S1-S3 and figure legends (without figures)

Table S4 Correlation between RCNs and driver mutations.

Figure S1 Flowchart showing the analysis pipeline used in the study.

Figure S2 Representative single channel images for each marker in the mIF panel.

Figure S3 Cell segmentation

Figure S4 Identification of recurrent cellular neighborhoods (RCNs) based on the number of nearest neighbors.

Figure S5 Proportions of immune cell subtypes in GCB and ABC DLBCL.

Figure S6 Proportions of immune cells subtypes in B2M, HLA-ABC, and HLA-DR positive and negative DLBCLs.

Figure S7 Clinical impact of immune cell subtypes in DLBCL NOS.

Figure S8 Cellular interactions in DLBCL NOS.

Figure S9 Proportion of B cells and T cells in lymphomas where B cells are avoiding and attracted to PD-1+ T cells.

Figure S10 Determining the optimal number of RCN clusters.

Figure S11 Proportions of cells with different RCNs in GCB and ABC DLBCLs.

Figure S12 Proportions of cells with different RCNs in B2M, HLA-ABC, and HLA-DR positive and negative DLBCLs.

Figure S13 Clinical impact of the distance between RCNs in DLBCL NOS.

Figure S14 Proportion of RCNs in lymphomas where these RCNs are situated close and far from each other.

Figure S15 Proportions of cells with different RCNs in DLBCLs with different lymphoma ecotypes (LE).

## References

[1] Sehn LH , Salles G. Diffuse large B-cell lymphoma. N Engl J Med 2021;384:842–58. 33657296 10.1056/NEJMra2027612 PMC8377611

[2] Westin JR , Oluwole OO, Kersten MJ, Miklos DB, Perales MA, Ghobadi A, . Survival with axicabtagene ciloleucel in large B-cell lymphoma. N Engl J Med 2023;389:148–57. 37272527 10.1056/NEJMoa2301665

[3] Falchi L , Vardhana SA, Salles GA. Bispecific antibodies for the treatment of B-cell lymphoma: promises, unknowns, and opportunities. Blood 2023;141:467–80. 36322929 10.1182/blood.2021011994 PMC9936308

[4] Alizadeh AA , Eisen MB, Davis RE, Ma C, Lossos IS, Rosenwald A, . Distinct types of diffuse large B-cell lymphoma identified by gene expression profiling. Nature 2000;403:503–11. 10676951 10.1038/35000501

[5] Schmitz R , Wright GW, Huang DW, Johnson CA, Phelan JD, Wang JQ, . Genetics and pathogenesis of diffuse large B-cell lymphoma. N Engl J Med 2018;378:1396–407. 29641966 10.1056/NEJMoa1801445 PMC6010183

[6] Chapuy B , Stewart C, Dunford AJ, Kim J, Kamburov A, Redd RA, . Molecular subtypes of diffuse large B cell lymphoma are associated with distinct pathogenic mechanisms and outcomes. Nat Med 2018;24:679–90. 29713087 10.1038/s41591-018-0016-8 PMC6613387

[7] Wright GW , Huang DW, Phelan JD, Coulibaly ZA, Roulland S, Young RM, . A probabilistic classification tool for genetic subtypes of diffuse large B cell lymphoma with therapeutic implications. Cancer Cell 2020;37:551–68.e14. 32289277 10.1016/j.ccell.2020.03.015 PMC8459709

[8] Kotlov N , Bagaev A, Revuelta MV, Phillip JM, Cacciapuoti MT, Antysheva Z, . Clinical and biological subtypes of B-cell lymphoma revealed by microenvironmental signatures. Cancer Discov 2021;11:1468–89. 33541860 10.1158/2159-8290.CD-20-0839 PMC8178179

[9] Steen CB , Luca BA, Esfahani MS, Azizi A, Sworder BJ, Nabet BY, . The landscape of tumor cell states and ecosystems in diffuse large B cell lymphoma. Cancer Cell 2021;39:1422–37.e10. 34597589 10.1016/j.ccell.2021.08.011 PMC9205168

[10] Scott DW , Gascoyne RD. The tumour microenvironment in B cell lymphomas. Nat Rev Cancer 2014;14:517–34. 25008267 10.1038/nrc3774

[11] Nicholas NS , Apollonio B, Ramsay AG. Tumor microenvironment (TME)-driven immune suppression in B cell malignancy. Biochim Biophys Acta 2016;1863:471–82. 26554850 10.1016/j.bbamcr.2015.11.003

[12] Gajewski TF , Schreiber H, Fu YX. Innate and adaptive immune cells in the tumor microenvironment. Nat Immunol 2013;14:1014–22. 24048123 10.1038/ni.2703 PMC4118725

[13] Cai QC , Liao H, Lin SX, Xia Y, Wang XX, Gao Y, . High expression of tumor-infiltrating macrophages correlates with poor prognosis in patients with diffuse large B-cell lymphoma. Med Oncol 2012;29:2317–22. 22198695 10.1007/s12032-011-0123-6

[14] Keane C , Gill D, Vari F, Cross D, Griffiths L, Gandhi M. CD4(+) tumor infiltrating lymphocytes are prognostic and independent of R-IPI in patients with DLBCL receiving R-CHOP chemo-immunotherapy. Am J Hematol 2013;88:273–6. 23460351 10.1002/ajh.23398

[15] Riihijärvi S , Fiskvik I, Taskinen M, Vajavaara H, Tikkala M, Yri O, . Prognostic influence of macrophages in patients with diffuse large B-cell lymphoma: a correlative study from a Nordic phase II trial. Haematologica 2015;100:238–45. 25381134 10.3324/haematol.2014.113472 PMC4803141

[16] Xu-Monette ZY , Xiao M, Au Q, Padmanabhan R, Xu B, Hoe N, . Immune profiling and quantitative analysis decipher the clinical role of immune-checkpoint expression in the tumor immune microenvironment of DLBCL. Cancer Immunol Res 2019;7:644–57. 30745366 10.1158/2326-6066.CIR-18-0439

[17] Autio M , Leivonen SK, Brück O, Mustjoki S, Mészáros Jørgensen J, Karjalainen-Lindsberg ML, . Immune cell constitution in the tumor microenvironment predicts the outcome in diffuse large B-cell lymphoma. Haematologica 2021;106:718–29. 32079690 10.3324/haematol.2019.243626 PMC7927991

[18] Autio M , Leivonen SK, Brück O, Karjalainen-Lindsberg ML, Pellinen T, Leppä S. Clinical impact of immune cells and their spatial interactions in diffuse large B-cell lymphoma microenvironment. Clin Cancer Res 2022;28:781–92. 34907083 10.1158/1078-0432.CCR-21-3140 PMC9377736

[19] Colombo AR , Hav M, Singh M, Xu A, Gamboa A, Lemos T, . Single-cell spatial analysis of tumor immune architecture in diffuse large B-cell lymphoma. Blood Adv 2022;6:4675–90. 35675517 10.1182/bloodadvances.2022007493 PMC9631676

[20] Wright KT , Weirather JL, Jiang S, Kao KZ, Sigal Y, Giobbie-Hurder A, . Diffuse large B-cell lymphomas have spatially defined, tumor immune microenvironments revealed by high-parameter imaging. Blood Adv 2023;7:4633–46. 37196647 10.1182/bloodadvances.2023009813 PMC10448427

[21] Reiss DJ , Nakayama Y, Weng AP, Stokes ME, Sehn L, Steidl C, . High-plex imaging and cellular neighborhood spatial analysis reveals multiple immune escape and suppression patterns in diffuse large B-cell lymphoma. Leukemia 2024;38:1164–8. 38575670 10.1038/s41375-024-02239-1 PMC11073958

[22] Liu WR , Shipp MA. Signaling pathways and immune evasion mechanisms in classical Hodgkin lymphoma. Blood 2017;130:2265–70. 29167175 10.1182/blood-2017-06-781989 PMC5701523

[23] Booman M , Douwes J, Glas AM, Riemersma SA, Jordanova ES, Kok K, . Mechanisms and effects of loss of human leukocyte antigen class II expression in immune-privileged site-associated B-cell lymphoma. Clin Cancer Res 2006;12:2698–705. 16675561 10.1158/1078-0432.CCR-05-2617

[24] Challa-Malladi M , Lieu YK, Califano O, Holmes AB, Bhagat G, Murty VV, . Combined genetic inactivation of β2-Microglobulin and CD58 reveals frequent escape from immune recognition in diffuse large B cell lymphoma. Cancer Cell 2011;20:728–40. 22137796 10.1016/j.ccr.2011.11.006 PMC3660995

[25] Rimsza LM , Roberts RA, Miller TP, Unger JM, LeBlanc M, Braziel RM, . Loss of MHC class II gene and protein expression in diffuse large B-cell lymphoma is related to decreased tumor immunosurveillance and poor patient survival regardless of other prognostic factors: a follow-up study from the Leukemia and Lymphoma Molecular Profiling Project. Blood 2004;103:4251–8. 14976040 10.1182/blood-2003-07-2365

[26] Steidl C , Shah SP, Woolcock BW, Rui L, Kawahara M, Farinha P, . MHC class II transactivator CIITA is a recurrent gene fusion partner in lymphoid cancers. Nature 2011;471:377–81. 21368758 10.1038/nature09754 PMC3902849

[27] Green MR , Monti S, Rodig SJ, Juszczynski P, Currie T, O'Donnell E, . Integrative analysis reveals selective 9p24.1 amplification, increased PD-1 ligand expression, and further induction via JAK2 in nodular sclerosing Hodgkin lymphoma and primary mediastinal large B-cell lymphoma. Blood 2010;116:3268–77. 20628145 10.1182/blood-2010-05-282780 PMC2995356

[28] Georgiou K , Chen L, Berglund M, Ren W, de Miranda NF, Lisboa S, . Genetic basis of PD-L1 overexpression in diffuse large B-cell lymphomas. Blood 2016;127:3026–34. 27030389 10.1182/blood-2015-12-686550

[29] Ansell SM , Lesokhin AM, Borrello I, Halwani A, Scott EC, Gutierrez M, . PD-1 blockade with nivolumab in relapsed or refractory Hodgkin's lymphoma. N Engl J Med 2015;372:311–9. 25482239 10.1056/NEJMoa1411087 PMC4348009

[30] Ansell SM , Minnema MC, Johnson P, Timmerman JM, Armand P, Shipp MA, . Nivolumab for relapsed/refractory diffuse large B-cell lymphoma in patients ineligible for or having failed autologous transplantation: a single-Arm, phase II study. J Clin Oncol 2019;37:481–9. 30620669 10.1200/JCO.18.00766 PMC6528729

[31] Nowakowski GS , Willenbacher W, Greil R, Larsen TS, Patel K, Jäger U, . Safety and efficacy of durvalumab with R-CHOP or R(2)-CHOP in untreated, high-risk DLBCL: a phase 2, open-label trial. Int J Hematol 2022;115:222–32. 34797531 10.1007/s12185-021-03241-4

[32] Nirmal AJ , Maliga Z, Vallius T, Quattrochi B, Chen AA, Jacobson CA, . The spatial landscape of progression and immunoediting in primary melanoma at single-cell resolution. Cancer Discov 2022;12:1518–41. 35404441 10.1158/2159-8290.CD-21-1357 PMC9167783

[33] Danenberg E , Bardwell H, Zanotelli VRT, Provenzano E, Chin SF, Rueda OM, . Breast tumor microenvironment structures are associated with genomic features and clinical outcome. Nat Genet 2022;54:660–9. 35437329 10.1038/s41588-022-01041-y PMC7612730

[34] Schürch CM , Bhate SS, Barlow GL, Phillips DJ, Noti L, Zlobec I, . Coordinated cellular neighborhoods orchestrate antitumoral immunity at the colorectal cancer invasive front. Cell 2020;182:1341–59.e19. 32763154 10.1016/j.cell.2020.07.005 PMC7479520

[35] Meriranta L , Pasanen A, Alkodsi A, Haukka J, Karjalainen-Lindsberg ML, Leppä S. Molecular background delineates outcome of double protein expressor diffuse large B-cell lymphoma. Blood Adv 2020;4:3742–53. 32780847 10.1182/bloodadvances.2020001727 PMC7422114

[36] Reddy A , Zhang J, Davis NS, Moffitt AB, Love CL, Waldrop A, . Genetic and functional drivers of diffuse large B cell lymphoma. Cell 2017;171:481–94.e15. 28985567 10.1016/j.cell.2017.09.027 PMC5659841

[37] Meriranta L , Sorri S, Huse K, Liu X, Spasevska I, Zafar S, . Disruption of KLHL6 fuels oncogenic antigen receptor signaling in B-cell lymphoma. Blood Cancer Discov 2024;5:331–52. 38630892 10.1158/2643-3230.BCD-23-0182 PMC11369598

[38] Blom S , Paavolainen L, Bychkov D, Turkki R, Mäki-Teeri P, Hemmes A, . Systems pathology by multiplexed immunohistochemistry and whole-slide digital image analysis. Sci Rep 2017;7:15580. 29138507 10.1038/s41598-017-15798-4 PMC5686230

[39] Hollandi R , Szkalisity A, Toth T, Tasnadi E, Molnar C, Mathe B, . nucleAIzer: a parameter-free deep learning framework for nucleus segmentation using image style transfer. Cell Syst 2020;10:453–8.e6. 34222682 10.1016/j.cels.2020.04.003 PMC8247631

[40] Schapiro D , Jackson HW, Raghuraman S, Fischer JR, Zanotelli VRT, Schulz D, . histoCAT: analysis of cell phenotypes and interactions in multiplex image cytometry data. Nat Methods 2017;14:873–6. 28783155 10.1038/nmeth.4391 PMC5617107

[41] Levine JH , Simonds EF, Bendall SC, Davis KL, Amir EaD, Tadmor MD, . Data-Driven phenotypic dissection of AML reveals progenitor-like cells that correlate with prognosis. Cell 2015;162:184–97. 26095251 10.1016/j.cell.2015.05.047 PMC4508757

[42] Blei DM , Ng AY, Jordan MI. Latent dirichlet allocation. J Mach Learn Res 2003;3:993–1022.

[43] Roider T , Baertsch MA, Fitzgerald D, Vöhringer H, Brinkmann BJ, Czernilofsky F, . Multimodal and spatially resolved profiling identifies distinct patterns of T cell infiltration in nodal B cell lymphoma entities. Nat Cell Biol 2024;26:478–89. 38379051 10.1038/s41556-024-01358-2 PMC10940160

[44] Fangazio M , Ladewig E, Gomez K, Garcia-Ibanez L, Kumar R, Teruya-Feldstein J, . Genetic mechanisms of HLA-I loss and immune escape in diffuse large B cell lymphoma. Proc Natl Acad Sci U S A 2021;118:e2104504118. 34050029 10.1073/pnas.2104504118 PMC8179151

[45] Dersh D , Phelan JD, Gumina ME, Wang B, Arbuckle JH, Holly J, . Genome-wide screens identify lineage- and tumor-specific genes modulating MHC-I- and MHC-II-restricted immunosurveillance of human lymphomas. Immunity 2021;54:116–31.e10. 33271120 10.1016/j.immuni.2020.11.002 PMC7874576

[46] O’Hayre M , Inoue A, Kufareva I, Wang Z, Mikelis CM, Drummond RA, . Inactivating mutations in GNA13 and RHOA in Burkitt’s lymphoma and diffuse large B-cell lymphoma: a tumor suppressor function for the Gα13/RhoA axis in B cells. Oncogene 2016;35:3771–80. 26616858 10.1038/onc.2015.442 PMC4885800

[47] Xia Y , Xu-Monette ZY, Tzankov A, Li X, Manyam GC, Murty V, . Loss of PRDM1/BLIMP-1 function contributes to poor prognosis of activated B-cell-like diffuse large B-cell lymphoma. Leukemia 2017;31:625–36. 27568520 10.1038/leu.2016.243 PMC5837859

