# Supplementary material for: Spatial Analysis of the Tumor Microenvironment in Diffuse Large B-cell Lymphoma Reveals Clinically Relevant Cell Interactions and Recurrent Cellular Neighborhoods
Source: Cancer Immunol Res. 2025 Aug 6;13(10):1674–86. doi: 10.1158/2326-6066.CIR-24-1163 (PMC12485370; doi:10.1158/2326-6066.CIR-24-1163)
Supplement: Tables S1-S3 — and figure legends (without figures) [file cir-24-1163_tables_s1-s3_suppst1-3.docx]

**Spatial Analysis of the Tumor Microenvironment in Diffuse Large B-Cell Lymphoma Reveals Clinically Relevant Cell Interactions and Recurrent Cellular Neighborhoods**

**Matias Autio et al.**

**Supplementary Materials**

**Supplementary Tables**

**Supplementary Table 1. The multiplex immunofluorescence panel with antibodies.**

| **1st round** |  |
| --- | --- |
| **TSA-488** | Alexa Fluor(R) 488 anti-T-bet (Biolegend; Cat# 644829, RRID:AB_2566018) 1:50 |
| **TSA-555** | Rabbit-anti-CD96 (Abcam; Cat# 264416, RRID:AB_3676524) 1:200 |
| **Alexa-647** | Rabbit-anti-PD-L1 (Cell Signaling Technologies; Cat# 13684, RRID:AB_2687655) 1:100 |
| **Alexa-750** | Mouse-anti-FOXP3 (Abcam; Cat# 20034, RRID:AB_445284) 1:100 |
|  | Bleach-boil |
| **2nd round** |  |
| **Alexa-647** | Rabbit-anti-CD4 (Abcam; Cat# ab133616, RRID:AB_2750883) 1:50 |
| **Alexa-750** | Mouse-anti-PD-1 (LSBio; Cat# LS-B12784, RRID:AB_3676525) 1:100 |
|  | Bleach-boil |
| **3rd round** |  |
| **Alexa-647** | Rabbit-anti-CD3 (Thermo Fischer Scientific; Cat# MA5-14482, RRID:AB_10985777) 1:400 |
| **Alexa-750** | Mouse-anti-CD8 (Agilent Dako; Cat# M7103, RRID:AB_2075537) 1:300 |
|  | Bleach-boil |
| **4th round** |  |
| **Alexa-647** | Mouse-anti-CD68 (Agilent Dako; Cat# M0876, RRID:AB_2074844) 1:50 |
| **Alexa-750** | Rabbit-anti-CD163 (Abcam; Cat# 188571, RRID:AB_3677451) 1:200 |
|  | Bleach-boil |
| **5th round** |  |
| **Alexa-647** | Mouse-anti-CD20 (Thermo Fischer Scientific; Cat# MS340-S1, RRID:AB_61493) 1:200 |
| **Alexa-750** | Rabbit-anti-CD45 (CST; Cat# 13917, RRID:AB_2750898) 1:100 |

**Supplementary Table 2. Patient characteristics for the groups with a TME where B cells are avoiding PD-1+ T cells and a TME where B cells are attracted to PD-1+ T cells**

| Characteristics | PD-1+ T cell – B cell avoidance n (%) | PD-1+ T cell – B cell attraction n (%) | *p*-val |
| --- | --- | --- | --- |
| No of patients | 50 (100) | 47 (100) |  |
| Age |  |  | 0.012 |
| <60 | 26 (52) | 12 (25) |  |
| ≥60 | 24 (48) | 35 (74) |  |
| Sex |  |  | 0.685 |
| Male | 30 (60) | 26 (55) |  |
| Female | 20 (40) | 21 (45) |  |
| Cell-of-origin |  |  | 0.022 |
| GCB* | 21 (42) | 10 (21) |  |
| ABC | 12 (24) | 23 (49) |  |
| Unclassified | 5 (10) | 4 (9) |  |
| nd | 12 (24) | 10 (21) |  |
| WHO PS |  |  | 0.172 |
| 0-1 | 40 (80) | 31 (66) |  |
| ≥2 | 10 (20) | 15 (32) |  |
| nd |  | 1 (2) |  |
| Stage |  |  | 0.148 |
| I-II | 33 (66) | 23 (49) |  |
| III-IV | 17 (34) | 23 (49) |  |
| nd |  | 1 (2) |  |
| IPI |  |  | 0.090 |
| 0-2 | 36 (72) | 24 (51) |  |
| 3-5 | 14 (28) | 20 (43) |  |
| nd |  | 3 (6) |  |
| Elevated LDH |  |  | 0.410 |
| Yes | 25 (50) | 27 (57) |  |
| No | 25 (50) | 18 (38) |  |
| nd |  | 2 (4) |  |
| EN |  |  | 0.398 |
| 0-1 | 44 (88) | 35 (74) |  |
| ≥2 | 6 (12) | 9 (19) |  |
| nd |  | 3 (6) |  |
| B-symptoms |  |  | 0.347 |
| Yes | 10 (20) | 14 (30) |  |
| No | 38 (76) | 32 (68) |  |
| nd | 2 (4) | 1 (2) |  |
| Inflamed TME |  |  | 0.014 |
| Yes | 21 (42) | 32 (68) |  |
| No | 29 (58) | 15 (32) |  |
| 5-year PFS | 83.4 % | 60.0 % |  |
| 5-year OS | 87.9 % | 63.5 % |  |

*GCB, germinal center B-cell like;

ABC, activated B-cell like;

nd, not determined;

IPI, international prognostic index;

LDH, lactate dehydrogenase;

EN, extranodal site;

R-CHOP, rituximab, cyclophosphamide, doxorubicin,

vincristine, prednisone;

R-ICE, rituximab, ifofamide, carboplatin, etoposide

**Supplementary Table 3. Average distances between different RCNs. The distances between cells in neighborhoods x and y are calculated as the average of the distance from cells in neighborhood x to cells in neighborhood y and the distance from cells in neighborhood y to cells in neighborhood x.**

| pixels | RCN1 | RCN2 | RCN3 | RCN4 | RCN5 | RCN6 | RCN7 | RCN8 | RCN9 | RCN10 |
| --- | --- | --- | --- | --- | --- | --- | --- | --- | --- | --- |
| RCN1 | 29.60 |  |  |  |  |  |  |  |  |  |
| RCN2 | 348.65 | 30.40 |  |  |  |  |  |  |  |  |
| RCN3 | 628.49 | 430.67 | 26.85 |  |  |  |  |  |  |  |
| RCN4 | 687.36 | 680.31 | 617.52 | 26.58 |  |  |  |  |  |  |
| RCN5 | 117.12 | 734.20 | 940.15 | 1023.73 | 27.81 |  |  |  |  |  |
| RCN6 | 522.72 | 508.03 | 661.34 | 969.54 | 786.30 | 31.84 |  |  |  |  |
| RCN7 | 757.44 | 529.86 | 960.69 | 686.80 | 1067.99 | 773.86 | 29.11 |  |  |  |
| RCN8 | 358.57 | 584.16 | 771.17 | 833.86 | 587.76 | 856.10 | 926.03 | 30.62 |  |  |
| RCN9 | 168.91 | 374.72 | 383.07 | 662.08 | 381.00 | 572.13 | 814.09 | 303.94 | 28.96 |  |
| RCN10 | 455.48 | 370.47 | 516.02 | 552.39 | 720.79 | 463.21 | 332.28 | 1003.62 | 520.81 | 30.24 |

**Supplementary Table 4. Correlation between RCNs and driver mutations.** Please see separate excel file.

**Supplementary Figure Legends**

**Supplementary Figure 1. Flowchart showing the analysis pipeline used in the study.**

mIF: multiplex immunofluorescence, TMA: tumor microarray

**Supplementary Figure 2. Representative single channel images for each marker in the mIF panel.**

Representative images of single channel images showing the staining of each anotbody used in the mIF panel. DAPI=gray. Scale bar 50 µm.

**Supplementary Figure 3. Cell segmentation**

Representative images of the cell segmentation masks overlaid on nuclei stained with DAPI in the mIF analyses performed on TMAs. The colored circles around the stained nuclei each represent the contours of a segmented cell. The colors are ramdom and do not represent different cell phenotypes. DAPI=gray.

**Supplementary Figure 4. Identification of recurrent cellular neighborhoods (RCNs) based on the number of nearest neighbors.**

Voronoi plots of three different TMA cores comparing the organization of cells into RCNs based on 10, 20, or 30 nearest neighbors to identify an RCN. Cells are colored based on their RCN. Colors in each plot are random.

**Supplementary Figure 5. Proportions of immune cell subtypes in GCB and ABC DLBCL.**

A-G) Boxplots depicting the proportion of B cells (A), CD4^+^ T cells (B), CD8^+^ T cells (C), Regulatory T cells (D), M1-like macrophages (E), M2-like macrophages (F), and non-immune cells (G) in GCB and ABC DLBCL as analyzed by mIHC. Statistical significance was analyzed using Mann-Whitney U test. TAMs: tumor associated macrophages.

**Supplementary Figure 6. Proportions of immune cells subtypes in B2M, HLA-ABC, and HLA-DR positive and negative DLBCLs.**

A-C) Boxplots depicting the proportions of immune cell subtypes in B2M (A), HLA-ABC (B), and HLA-DR (C) negative and positive DLBCLs analyzed by mIHC. Statistical significance was analyzed using Mann-Whitney U test. TAMs: tumor associated macrophages.

**Supplementary Figure 7. Clinical impact of immune cell subtypes in DLBCL NOS.**

A-B) Forest plots visualizing the impact of the identified immune cell subtypes on OS (A) and PFS (B) in DLBCL NOS patients treated with R-CHOP like immunochemotherapy, as evaluated by Cox univariable regression analyses with continuous variables. TAMs: tumor associated macrophages.

**Supplementary Figure 8. Cellular interactions in DLBCL NOS.**

A) Unsupervised hierarchical clustering of all the studied cell interactions in all patient samples. Cell interactions are depicted on the y-axis and patients on the x-axis. Red color indicates attraction and blue color avoidance between the studied cell types. TAMs: tumor associated macrophages.

**Supplementary Figure 9. Proportion of B cells and T cells in lymphomas where B cells are avoiding and attracted to PD-1^+^ T cells.**

Violin plots depicting the proportion of B cells, CD4^+^ T cells, PD-1^+^ CD4^+^ T cells, CD8^+^ T cells, and PD-1^+^ CD8^+^ T cells in lymphomas where B cells are avoiding and attracted to PD-1^+^ T cells as defined using unsupervised hierarchical clustering in Figure 2B.

**Supplementary Figure 10. Determining the optimal number of RCN clusters.**

A) A scree plot showing the decrease of within sum of squares with an increasing number of K-means clusters.

**Supplementary Figure 11. Proportions of cells with different RCNs in GCB and ABC DLBCLs.**

A) A heatmap depicting the average proportions of cells with B cell rich with immune cells RCN1, CD8^+^ T cell rich RCN2, CD4^+^ T cell rich RCN3, PD-1^+^ cell rich RCN4, immune poor RCN5, M2-like macrophage/non-immune cell rich RCN6, PD-L1^+^ B cell rich RCN7, M1-like macrophage rich RCN8, B-cell rich with T cells RCN9, and PD-L1+ M2-like macrophage rich RCN10 neighborhoods in GCB and ABC DLBCLs.

**Supplementary Figure 12. Proportions of cells with different RCNs in B2M, HLA-ABC, and HLA-DR positive and negative DLBCLs.**

A-B) Heatmaps depicting the average proportions depicting the average proportions of cells with B cell rich with immune cells RCN1, CD8^+^ T cell rich RCN2, CD4^+^ T cell rich RCN3, PD-1^+^ cell rich RCN4, immune poor RCN5, M2-like macrophage/non-immune cell rich RCN6, PD-L1^+^ B cell rich RCN7, M2-like macrophage rich RCN8, B-cell rich with T cells RCN9, and PD-L1^+^ M2-like macrophage rich RCN10 neighborhoods in B2M (A) and HLA-ABC (B) positive and negative DLBCLs.

C-D) Boxplots depicting the proportions of cells with RCN1 (C) and RCN4 (D) neighborhoods in HLA-ABC positive and negative DLBCLs.

E) A heatmap depicting the proportions of cells with RCN1-RCN10 neighborhoods in HLA-DR positive and negative DLBCLs.

F-G) Boxplots depicting the proportions of cells with RCN6 (F) and RCN9 (G) neighborhoods in HLA-DR positive and negative DLBCLs.

**Supplementary Figure 13. Clinical impact of the distance between RCNs in DLBCL NOS.**

A) Kaplan-Meier (log-rank test) survival plot depicts OS in R-CHOP treated DLBCL NOS patients in whose lymphomas CD8^+^ T cell rich RCN2 neighborhoods are situated very close to immune poor RCN5 neighborhoods and in patients in whose lymphomas these RCNs are situated far from each other or are not present.

B) Kaplan-Meier (log-rank test) survival plot depicts OS in R-CHOP treated DLBCL NOS patients in whose lymphomas PD-L1^+^ B cell rich RCN7 neighborhoods are situated very close or close to CD8^+^ T cell rich RCN2 neighborhoods and in patients in whose lymphomas these RCNs are situated far from each other or are not present.

C) A forest plot visualizing the impact of the proximity of PD-L1^+^ B cell rich RCN7 neighborhoods to CD8^+^ T cell rich RCN2 neighborhoods on OS in a Cox multivariable regression analysis with IPI and COO in R-CHOP treated DLBCL NOS patients.

D-E) Kaplan-Meier (log-rank test) survival plots depict OS (D) and PFS (E) in R-CHOP treated DLBCL NOS patients in whose lymphomas PD-L1^+^ M2-like macrophage rich RCN10 neighborhoods are situated very close to CD8^+^ T cell rich RCN2 neighborhoods and in patients in whose lymphomas these RCNs are situated far from each other or not present.

F) A forest-plot visualizing the impact of the proximity of PD-L1^+^ M2-like macrophage rich RCN10 neighborhoods to CD8^+^ T cell rich RCN2 neighborhoods on OS in a Cox multivariable regression analysis with IPI and COO in R-CHOP treated DLBCL NOS patients.

**Supplementary Figure 14. Proportion of RCNs in lymphomas where these RCNs are situated close and far from each other.**

A) Violin plots depicting the proportion of CD8^+^ T cell rich RCN2 and immune poor RCN5 in lymphomas where these RCNs are situated close as well as far from each other.

B) Violin plots depicting the proportion of CD8^+^ T cell rich RCN2 and PD-L1^+^ B cell rich RCN7 in lymphomas where these RCNs are situated close as well as far from each other.

**Supplementary Figure 15. Proportions of cells with different RCNs in DLBCLs with different lymphoma ecotypes (LE).**

A) A heatmap depicting the average proportions of cells with B cell rich with immune cells RCN1, CD8^+^ T cell rich RCN2, CD4^+^ T cell rich RCN3, PD-1^+^ cell rich RCN4, immune poor RCN5, M2-like macrophage/non-immune cell rich RCN6, PD-L1^+^ B cell rich RCN7, M1-like macrophage rich RCN8, B-cell rich with T cells RCN9, and PD-L1^+^ M2-like macrophage rich RCN10 neighborhoods in DLBCLs with LE1-LE9.
