# Supplementary material for: Spatial Analysis of the Tumor Microenvironment in Diffuse Large B-cell Lymphoma Reveals Clinically Relevant Cell Interactions and Recurrent Cellular Neighborhoods
Source: Cancer Immunol Res. 2025 Aug 6;13(10):1674–86. doi: 10.1158/2326-6066.CIR-24-1163 (PMC12485370; doi:10.1158/2326-6066.CIR-24-1163)
Supplement: Figure S1 — Flowchart showing the analysis pipeline used in the study. [file cir-24-1163_figure_s1_supps1.docx]

**Supplementary Figure 1. Flowchart showing the analysis pipeline used in the study.**

**
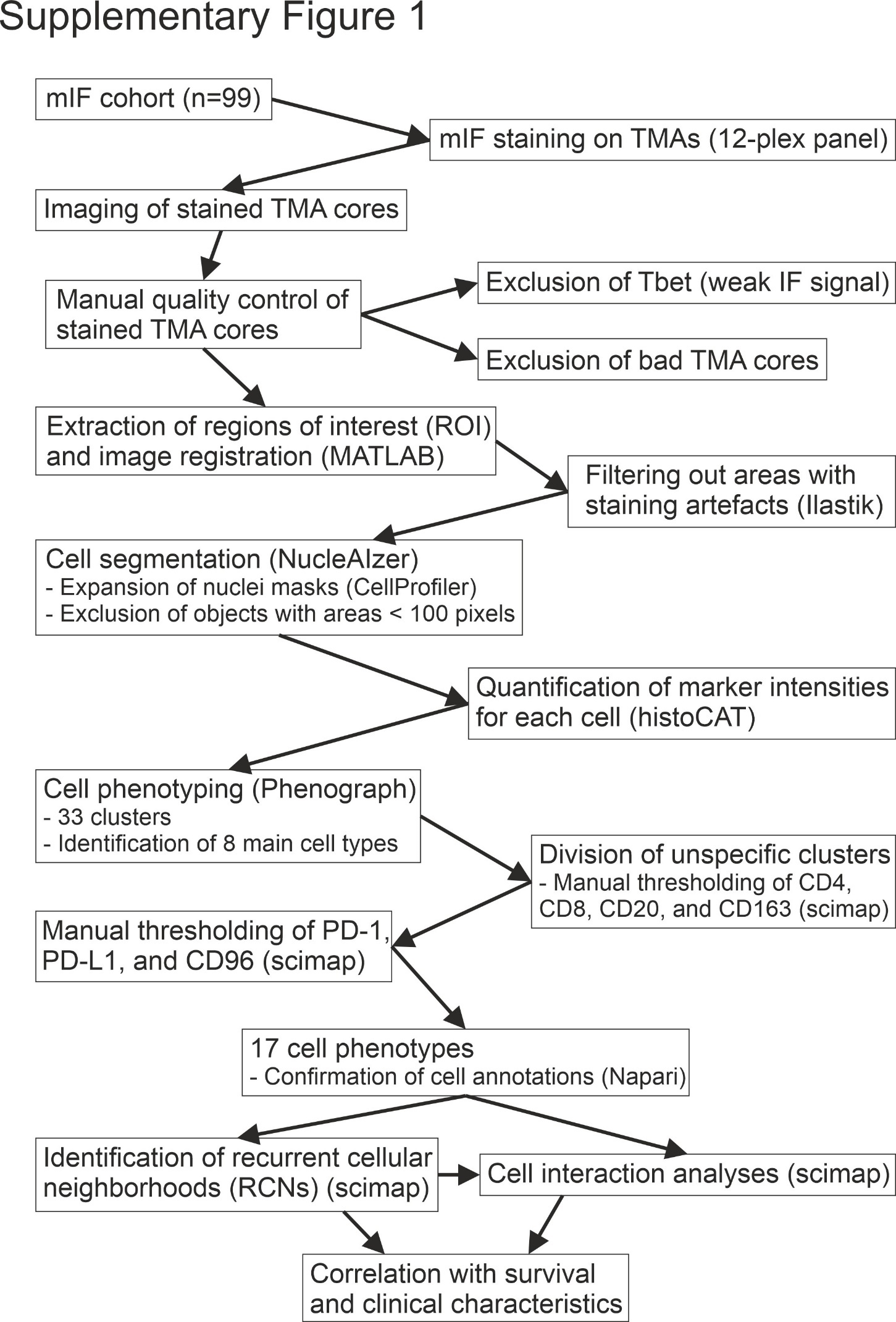
**

**Supplementary Figure 1. Flowchart showing the analysis pipeline used in the study.**

mIF: multiplex immunofluorescence, TMA: tumor microarray
