# Supplementary material for: Spatial Analysis of the Tumor Microenvironment in Diffuse Large B-cell Lymphoma Reveals Clinically Relevant Cell Interactions and Recurrent Cellular Neighborhoods
Source: Cancer Immunol Res. 2025 Aug 6;13(10):1674–86. doi: 10.1158/2326-6066.CIR-24-1163 (PMC12485370; doi:10.1158/2326-6066.CIR-24-1163)
Supplement: Figure S2 — Representative single channel images for each marker in the mIF panel. [file cir-24-1163_figure_s2_supps2.docx]

**Supplementary Figure 2.** **Representative single channel images for each marker in the mIF panel.**

**
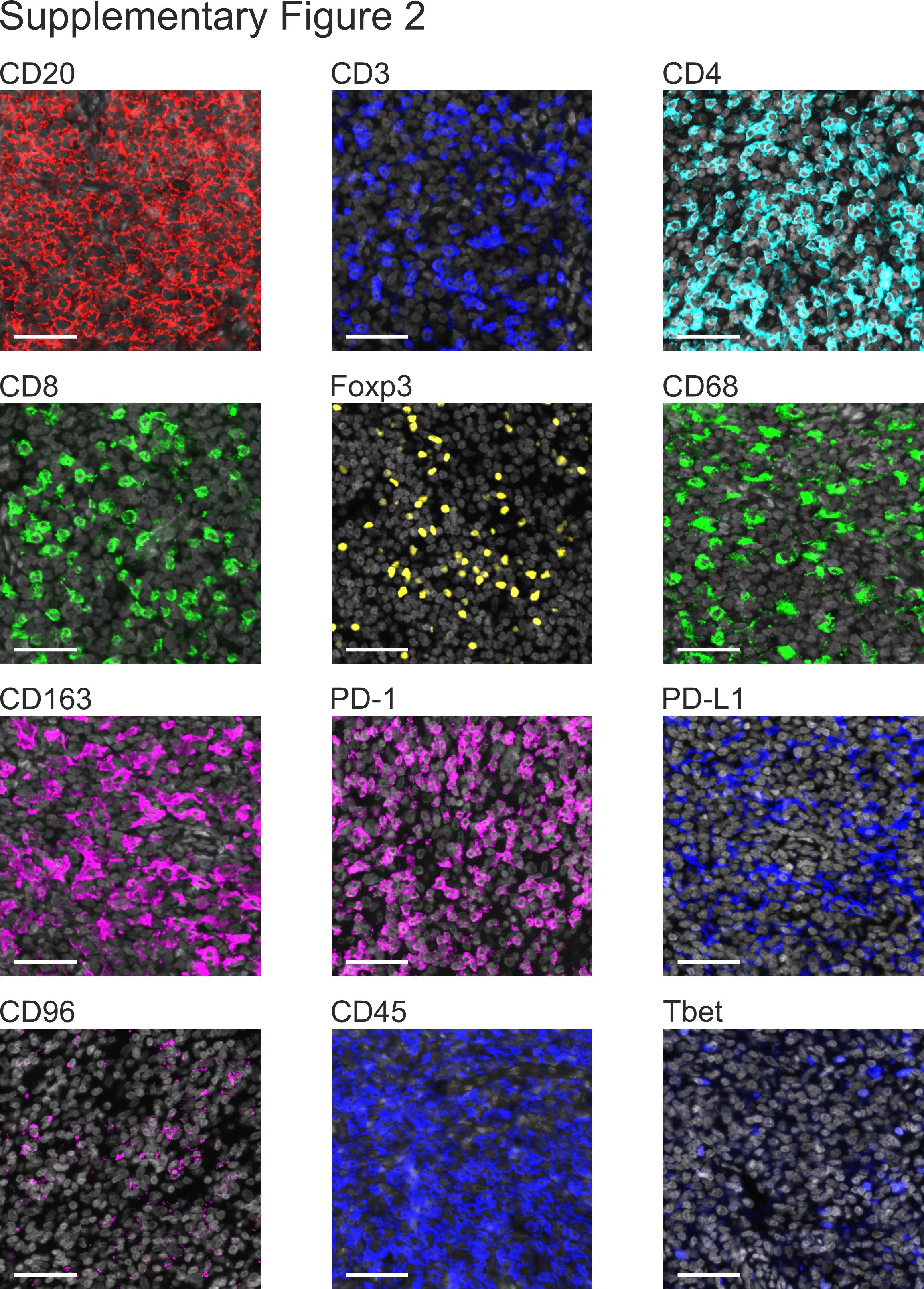
**

**Supplementary Figure 2. Representative single channel images for each marker in the mIF panel.**

Representative images of single channel images showing the staining of each anotbody used in the mIF panel. DAPI=gray. Scale bar 50 µm.
