# Supplementary material for: Spatial Analysis of the Tumor Microenvironment in Diffuse Large B-cell Lymphoma Reveals Clinically Relevant Cell Interactions and Recurrent Cellular Neighborhoods
Source: Cancer Immunol Res. 2025 Aug 6;13(10):1674–86. doi: 10.1158/2326-6066.CIR-24-1163 (PMC12485370; doi:10.1158/2326-6066.CIR-24-1163)
Supplement: Figure S3 — Cell segmentation [file cir-24-1163_figure_s3_supps3.docx]

**Supplementary Figure 3. Cell segmentation**

**
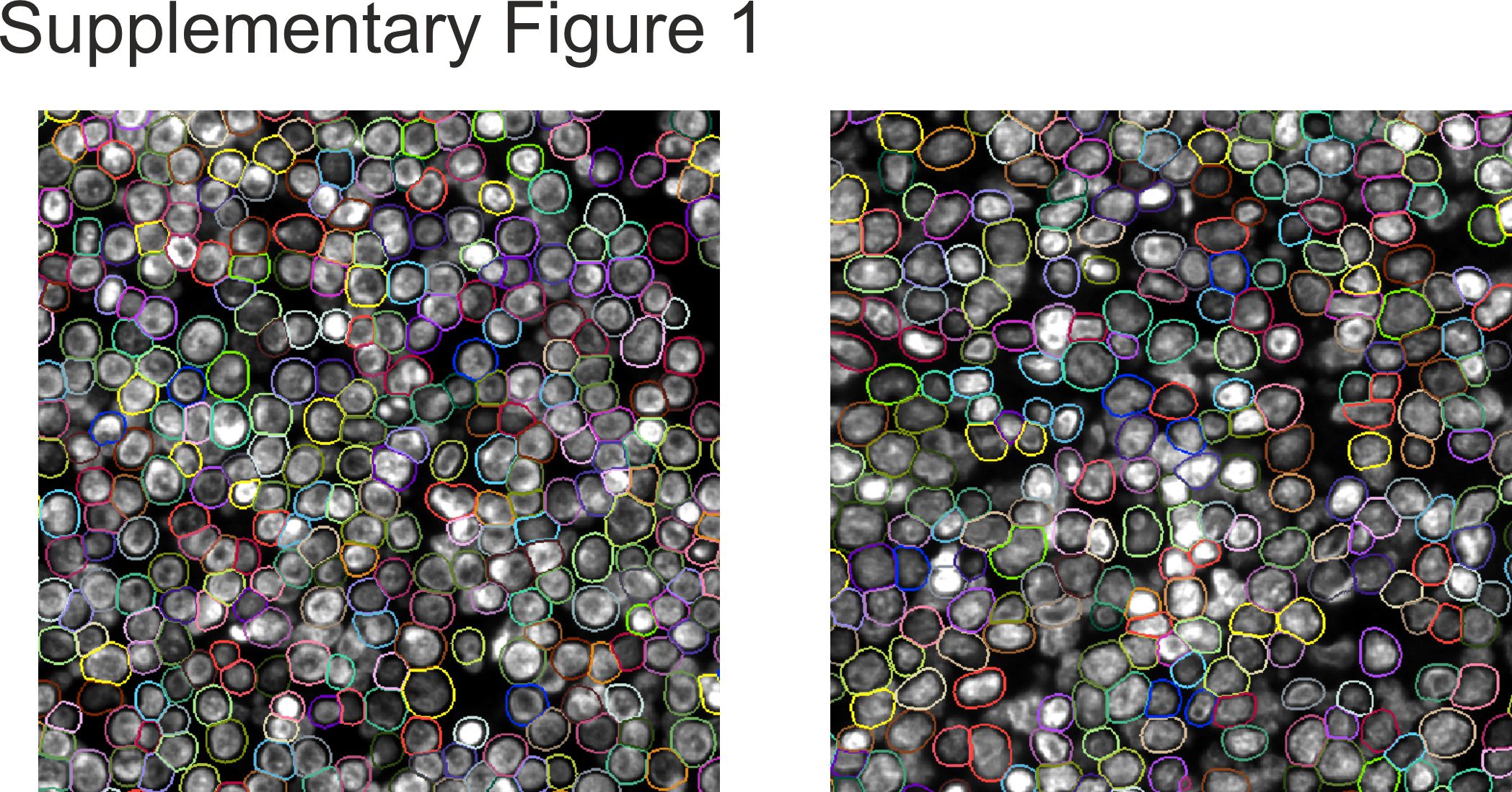
**

**Supplementary Figure 3. Cell segmentation**

Representative images of the cell segmentation masks overlaid on nuclei stained with DAPI in the mIF analyses performed on TMAs. The colored circles around the stained nuclei each represent the contours of a segmented cell. The colors are ramdom and do not represent different cell phenotypes. DAPI=gray.
