# Supplementary material for: Spatial Analysis of the Tumor Microenvironment in Diffuse Large B-cell Lymphoma Reveals Clinically Relevant Cell Interactions and Recurrent Cellular Neighborhoods
Source: Cancer Immunol Res. 2025 Aug 6;13(10):1674–86. doi: 10.1158/2326-6066.CIR-24-1163 (PMC12485370; doi:10.1158/2326-6066.CIR-24-1163)
Supplement: Figure S4 — Identification of recurrent cellular neighborhoods (RCNs) based on the number of nearest neighbors. [file cir-24-1163_figure_s4_supps4.docx]

**Supplementary Figure 4. Identification of recurrent cellular neighborhoods (RCNs) based on the number of nearest neighbors.**

**
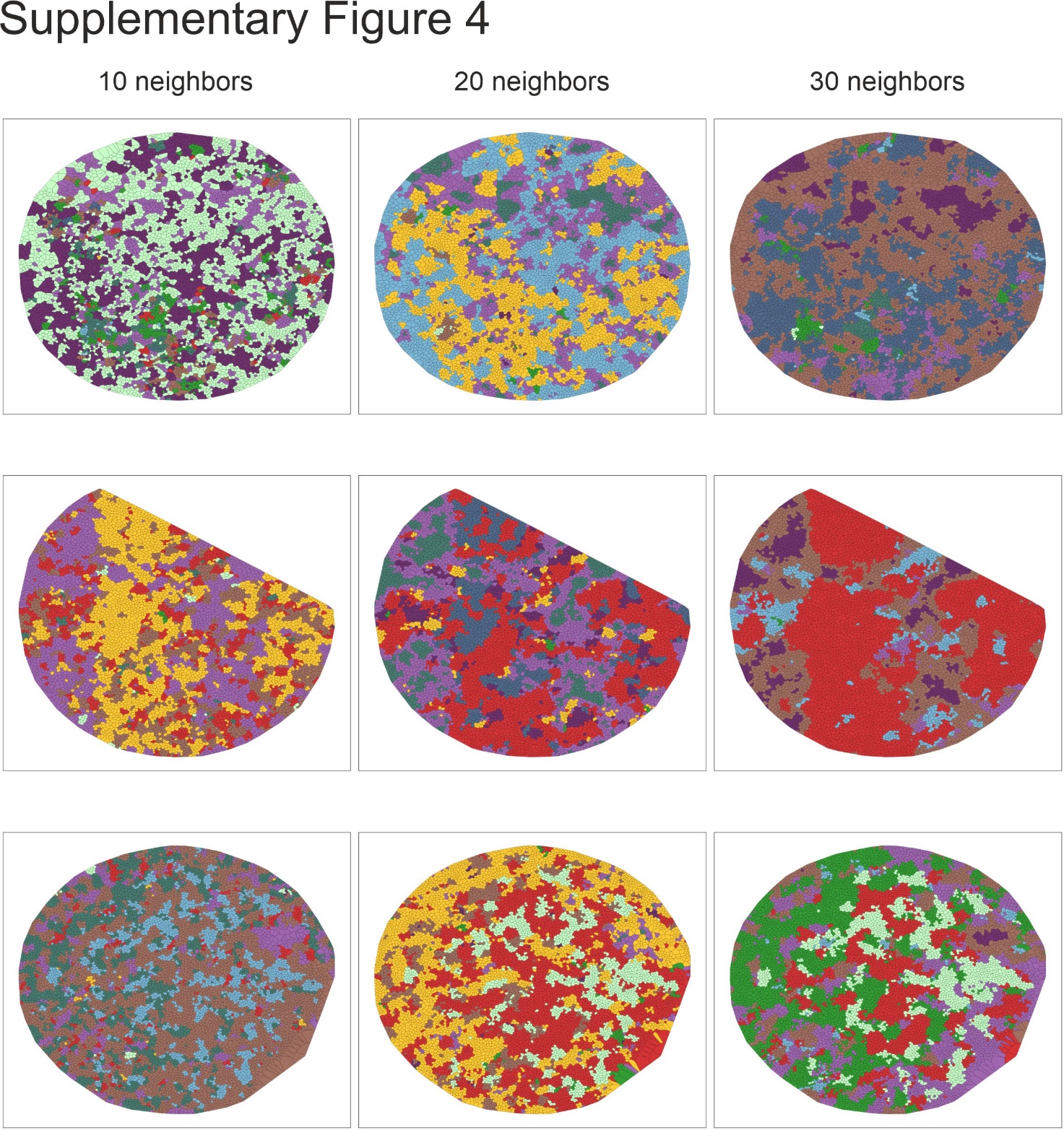
**

**Supplementary Figure 4. Identification of recurrent cellular neighborhoods (RCNs) based on the number of nearest neighbors.**

Voronoi plots of three different TMA cores comparing the organization of cells into RCNs based on 10, 20, or 30 nearest neighbors to identify an RCN. Cells are colored based on their RCN. Colors in each plot are random.
