# Supplementary material for: Spatial Analysis of the Tumor Microenvironment in Diffuse Large B-cell Lymphoma Reveals Clinically Relevant Cell Interactions and Recurrent Cellular Neighborhoods
Source: Cancer Immunol Res. 2025 Aug 6;13(10):1674–86. doi: 10.1158/2326-6066.CIR-24-1163 (PMC12485370; doi:10.1158/2326-6066.CIR-24-1163)
Supplement: Figure S5 — Proportions of immune cell subtypes in GCB and ABC DLBCL. [file cir-24-1163_figure_s5_supps5.docx]

**Supplementary Figure 5. Proportions of immune cell subtypes in GCB and ABC DLBCL.**

**
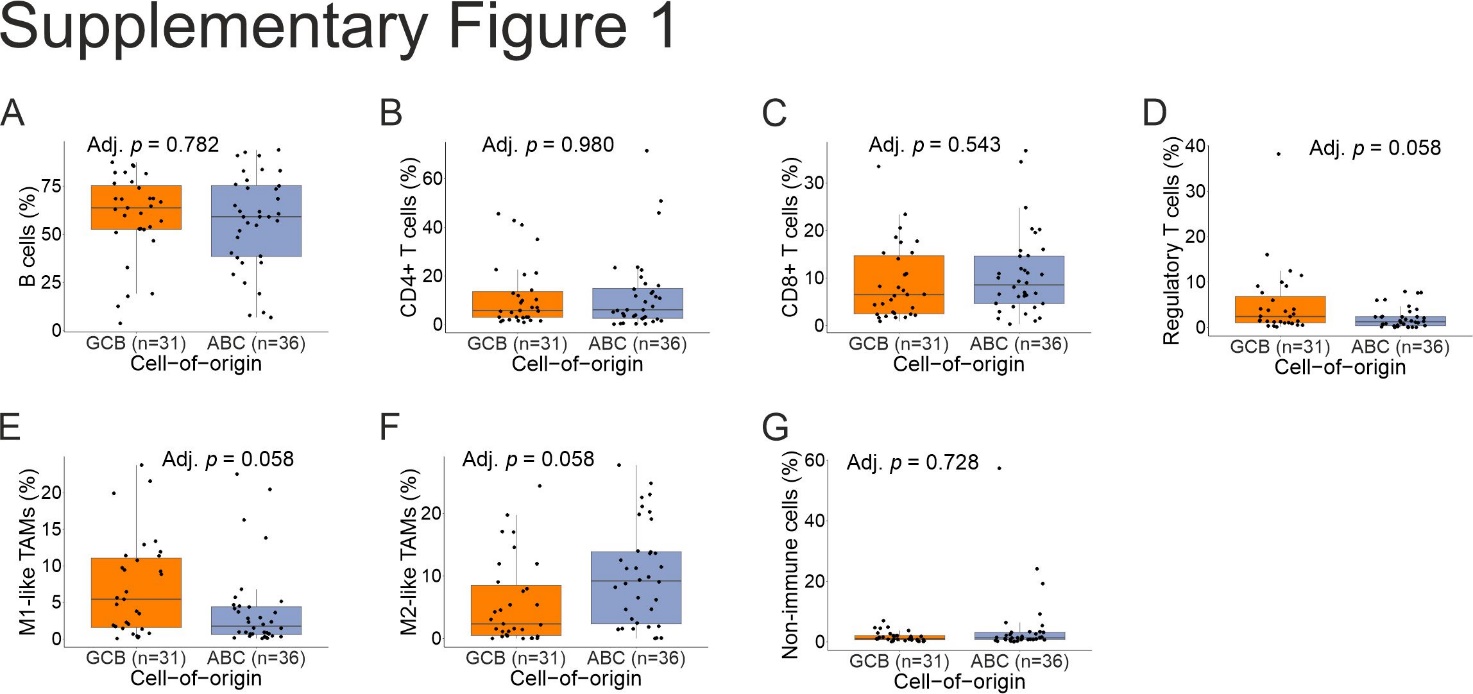
**

**Supplementary Figure 5. Proportions of immune cell subtypes in GCB and ABC DLBCL.**

A-G) Boxplots depicting the proportion of B cells (A), CD4^+^ T cells (B), CD8^+^ T cells (C), Regulatory T cells (D), M1-like macrophages (E), M2-like macrophages (F), and non-immune cells (G) in GCB and ABC DLBCL as analyzed by mIHC. Statistical significance was analyzed using Mann-Whitney U test. TAMs: tumor associated macrophages.
