# Supplementary material for: Spatial Analysis of the Tumor Microenvironment in Diffuse Large B-cell Lymphoma Reveals Clinically Relevant Cell Interactions and Recurrent Cellular Neighborhoods
Source: Cancer Immunol Res. 2025 Aug 6;13(10):1674–86. doi: 10.1158/2326-6066.CIR-24-1163 (PMC12485370; doi:10.1158/2326-6066.CIR-24-1163)
Supplement: Figure S6 — Proportions of immune cells subtypes in B2M, HLA-ABC, and HLA-DR positive and negative DLBCLs. [file cir-24-1163_figure_s6_supps6.docx]

**Supplementary Figure 6. Proportions of immune cells subtypes in B2M, HLA-ABC, and HLA-DR positive and negative DLBCLs.**

**
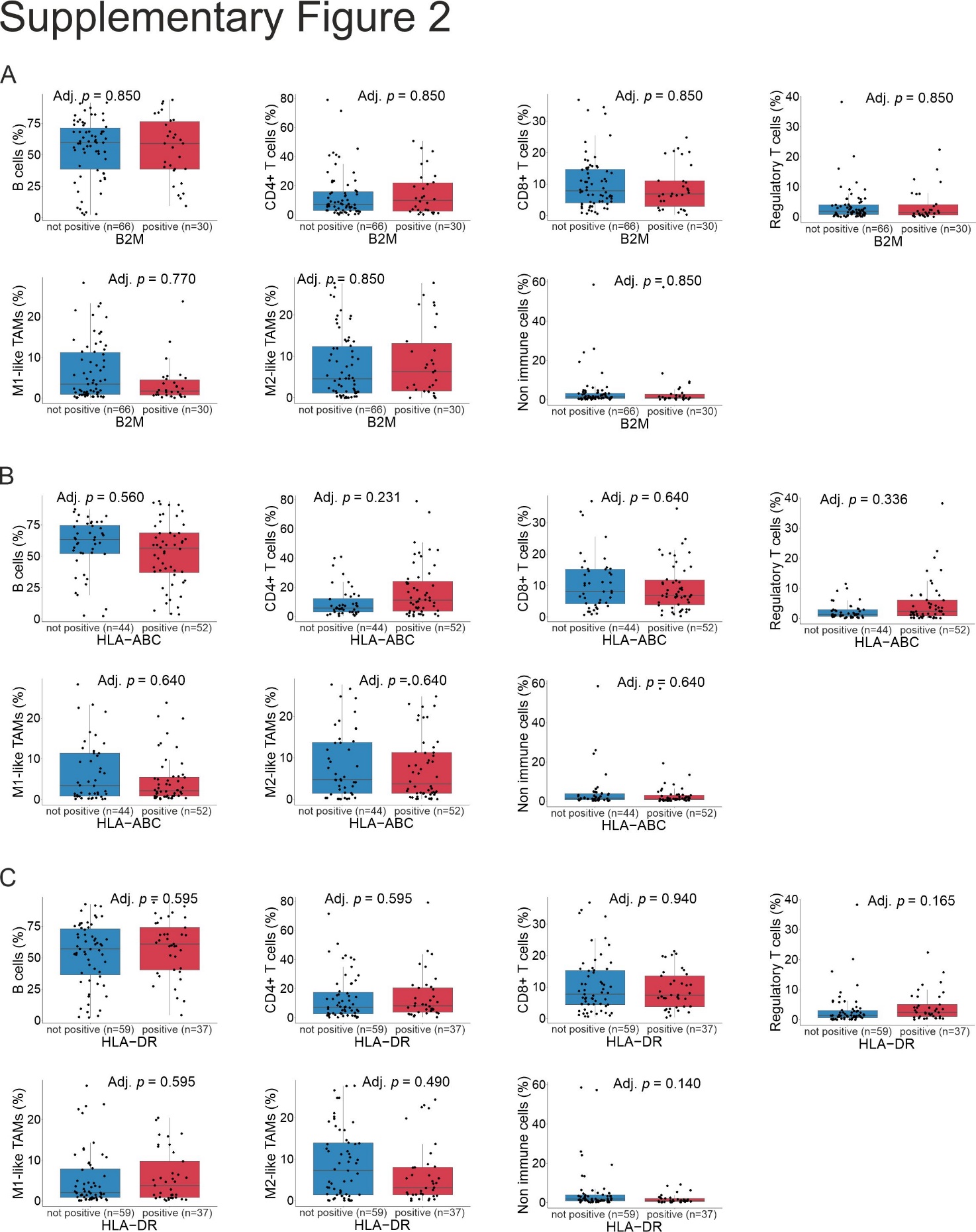
**

**Supplementary Figure 6. Proportions of immune cells subtypes in B2M, HLA-ABC, and HLA-DR positive and negative DLBCLs.**

A-C) Boxplots depicting the proportions of immune cell subtypes in B2M (A), HLA-ABC (B), and HLA-DR (C) negative and positive DLBCLs analyzed by mIHC. Statistical significance was analyzed using Mann-Whitney U test. TAMs: tumor associated macrophages.
