# Supplementary material for: Spatial Analysis of the Tumor Microenvironment in Diffuse Large B-cell Lymphoma Reveals Clinically Relevant Cell Interactions and Recurrent Cellular Neighborhoods
Source: Cancer Immunol Res. 2025 Aug 6;13(10):1674–86. doi: 10.1158/2326-6066.CIR-24-1163 (PMC12485370; doi:10.1158/2326-6066.CIR-24-1163)
Supplement: Figure S7 — Clinical impact of immune cell subtypes in DLBCL NOS. [file cir-24-1163_figure_s7_supps7.docx]

**Supplementary Figure 7. Clinical impact of immune cell subtypes in DLBCL NOS.**

**
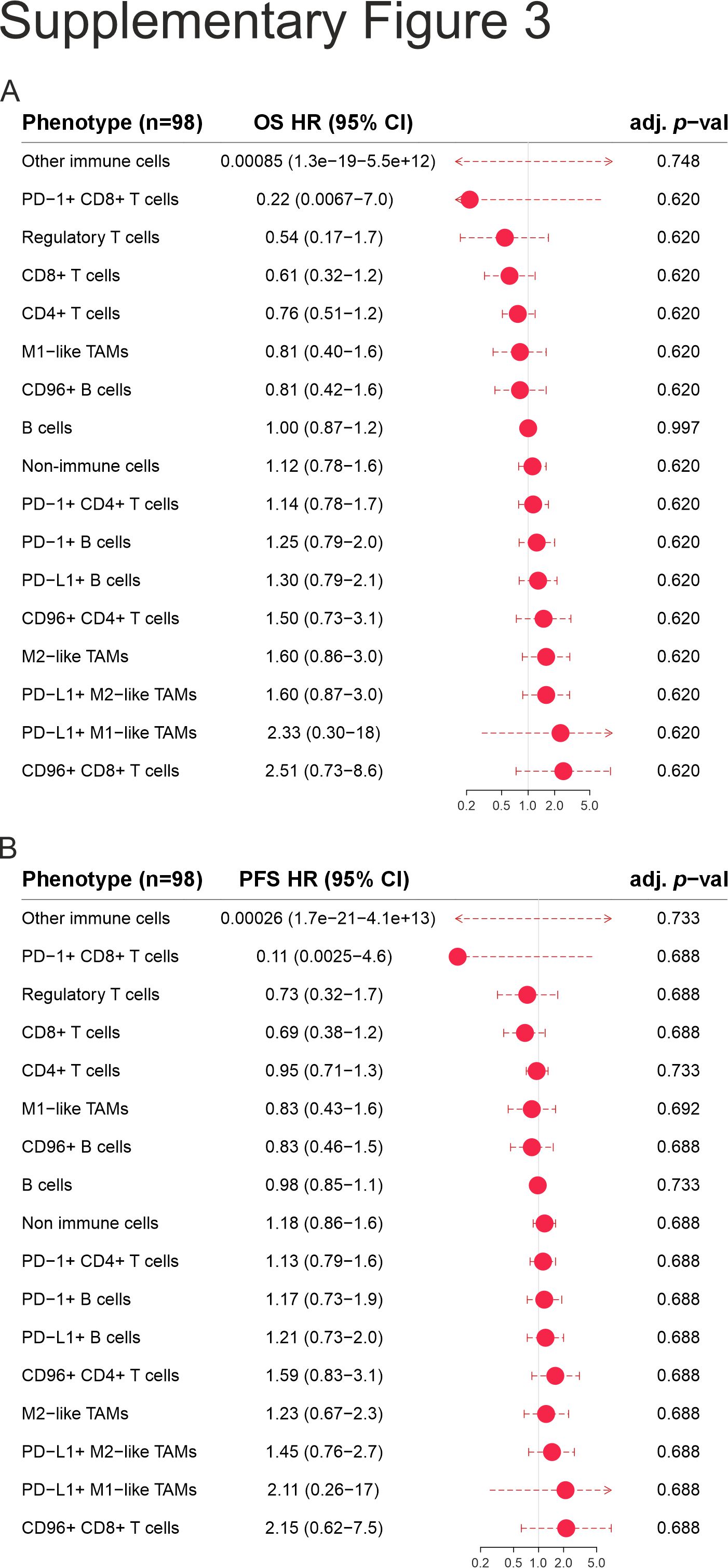
**

**Supplementary Figure 7. Clinical impact of immune cell subtypes in DLBCL NOS.**

A-B) Forest plots visualizing the impact of the identified immune cell subtypes on OS (A) and PFS (B) in DLBCL NOS patients treated with R-CHOP like immunochemotherapy, as evaluated by Cox univariable regression analyses with continuous variables. TAMs: tumor associated macrophages.
