# Supplementary material for: Spatial Analysis of the Tumor Microenvironment in Diffuse Large B-cell Lymphoma Reveals Clinically Relevant Cell Interactions and Recurrent Cellular Neighborhoods
Source: Cancer Immunol Res. 2025 Aug 6;13(10):1674–86. doi: 10.1158/2326-6066.CIR-24-1163 (PMC12485370; doi:10.1158/2326-6066.CIR-24-1163)
Supplement: Figure S8 — Cellular interactions in DLBCL NOS. [file cir-24-1163_figure_s8_supps8.docx]

**Supplementary Figure 8. Cellular interactions in DLBCL NOS.**


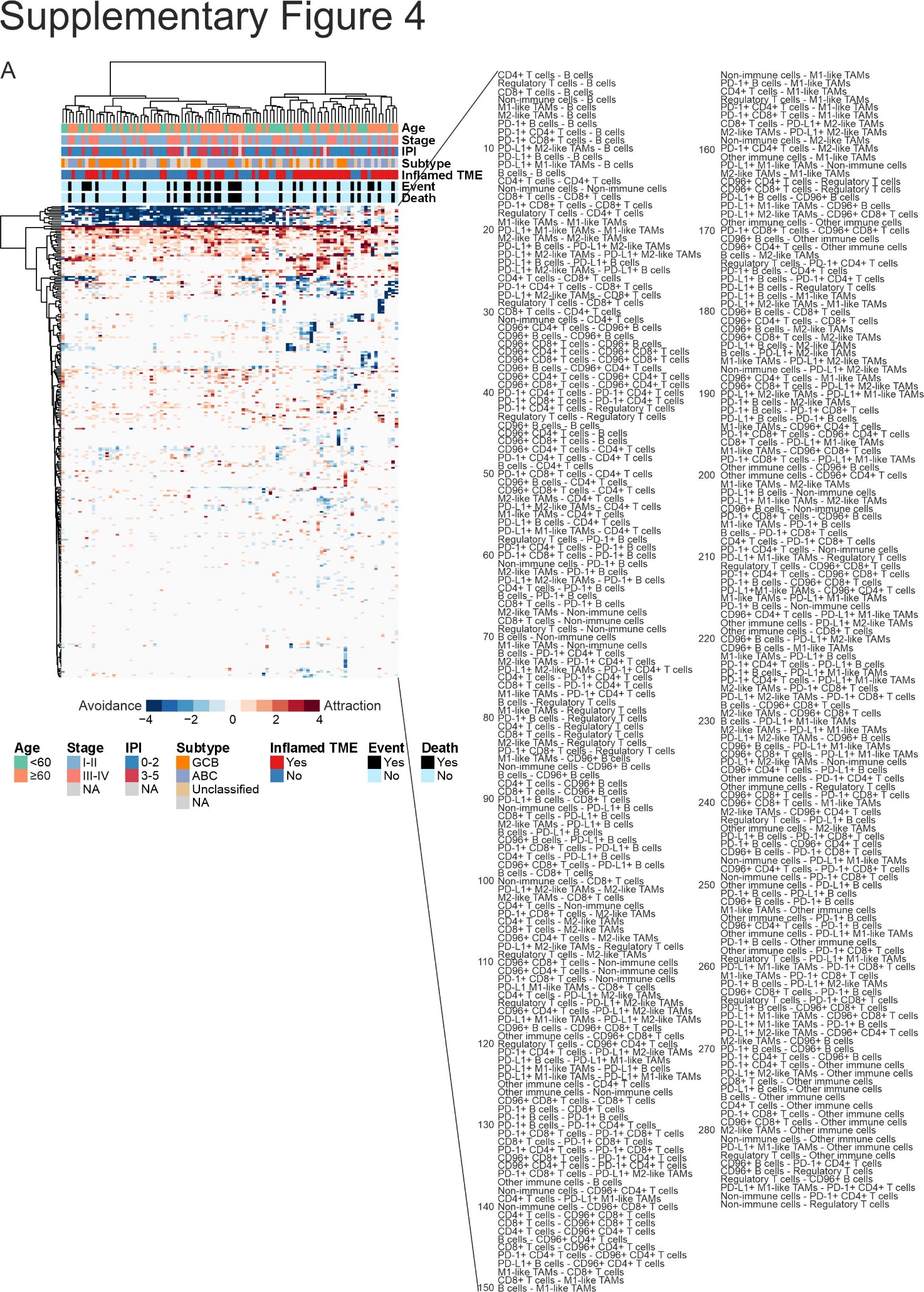


**Supplementary Figure 8. Cellular interactions in DLBCL NOS.**

A) Unsupervised hierarchical clustering of all the studied cell interactions in all patient samples. Cell interactions are depicted on the y-axis and patients on the x-axis. Red color indicates attraction and blue color avoidance between the studied cell types. TAMs: tumor associated macrophages.
