# Supplementary material for: Spatial Analysis of the Tumor Microenvironment in Diffuse Large B-cell Lymphoma Reveals Clinically Relevant Cell Interactions and Recurrent Cellular Neighborhoods
Source: Cancer Immunol Res. 2025 Aug 6;13(10):1674–86. doi: 10.1158/2326-6066.CIR-24-1163 (PMC12485370; doi:10.1158/2326-6066.CIR-24-1163)
Supplement: Figure S9 — Proportion of B cells and T cells in lymphomas where B cells are avoiding and attracted to PD-1+ T cells. [file cir-24-1163_figure_s9_supps9.docx]

**Supplementary Figure 9. Proportion of B cells and T cells in lymphomas where B cells are avoiding and attracted to PD-1^+^ T cells.**

**
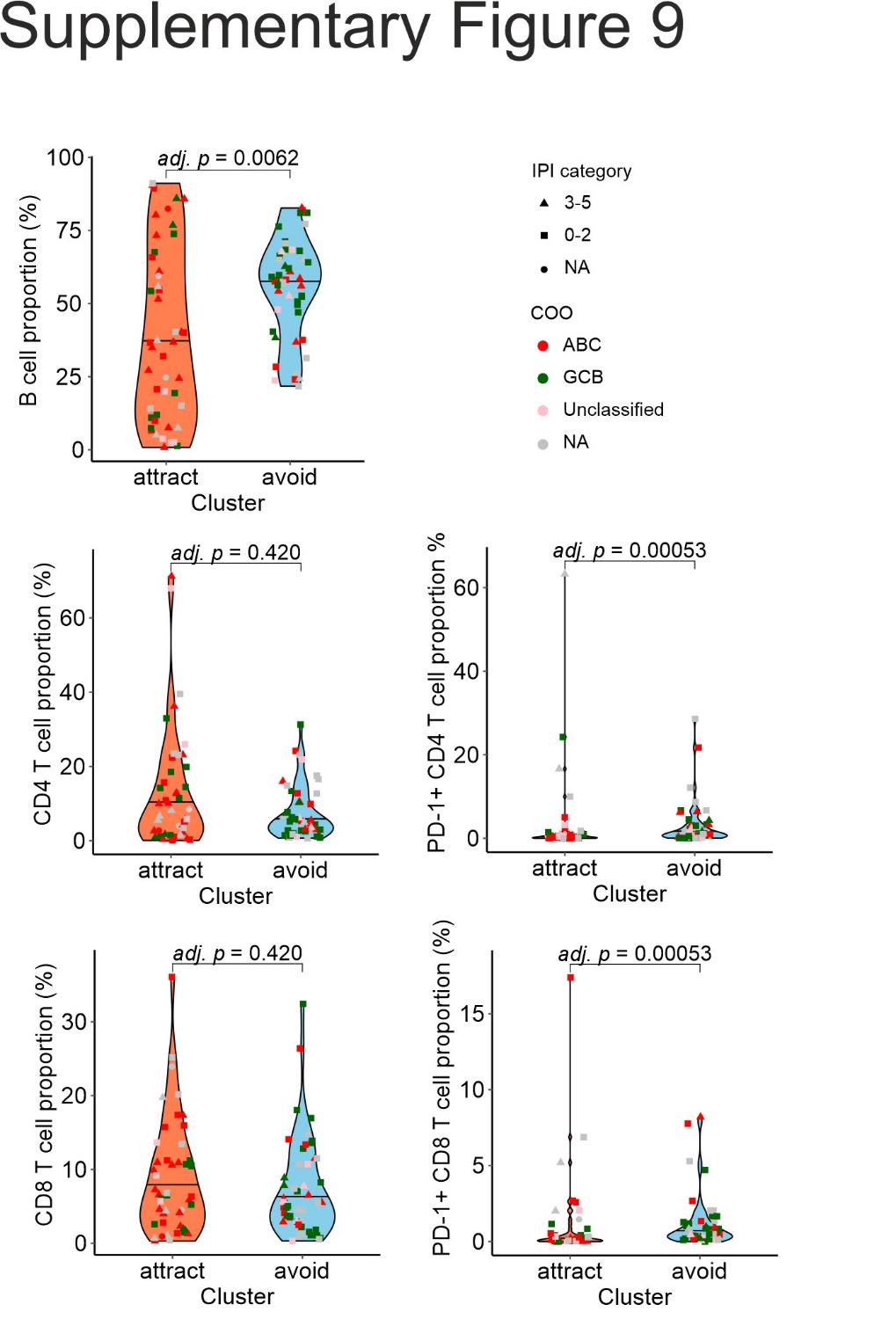
**

**Supplementary Figure 9. Proportion of B cells and T cells in lymphomas where B cells are avoiding and attracted to PD-1^+^ T cells.**

Violin plots depicting the proportion of B cells, CD4^+^ T cells, PD-1^+^ CD4^+^ T cells, CD8^+^ T cells, and PD-1^+^ CD8^+^ T cells in lymphomas where B cells are avoiding and attracted to PD-1^+^ T cells as defined using unsupervised hierarchical clustering in Figure 2B.
