# Supplementary material for: Spatial Analysis of the Tumor Microenvironment in Diffuse Large B-cell Lymphoma Reveals Clinically Relevant Cell Interactions and Recurrent Cellular Neighborhoods
Source: Cancer Immunol Res. 2025 Aug 6;13(10):1674–86. doi: 10.1158/2326-6066.CIR-24-1163 (PMC12485370; doi:10.1158/2326-6066.CIR-24-1163)
Supplement: Figure S10 — Determining the optimal number of RCN clusters. [file cir-24-1163_figure_s10_supps10.docx]

**Supplementary Figure 10. Determining the optimal number of RCN clusters.**


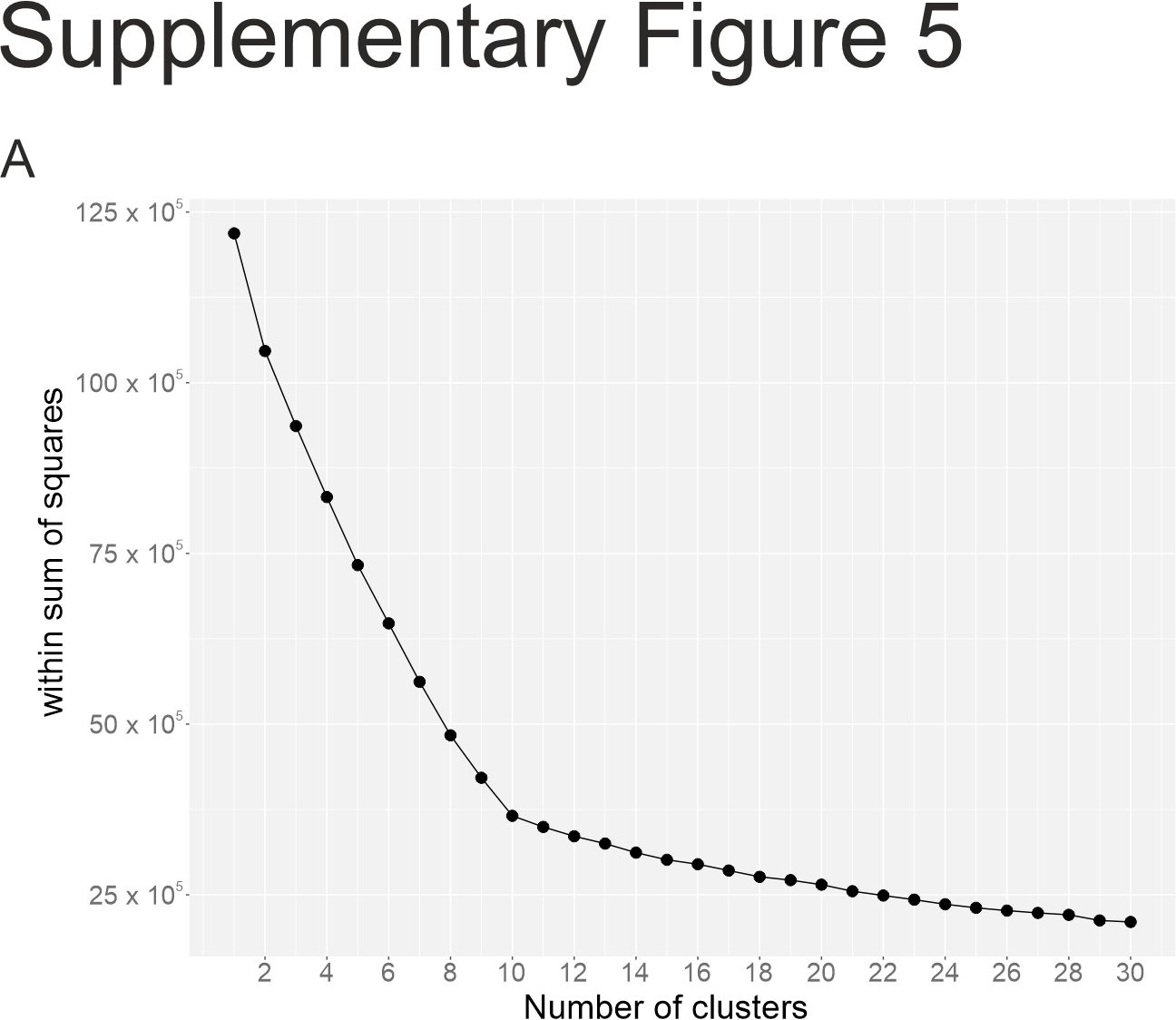


**Supplementary Figure 10. Determining the optimal number of RCN clusters.**

A) A scree plot showing the decrease of within sum of squares with an increasing number of K-means clusters.
