# Supplementary material for: Spatial Analysis of the Tumor Microenvironment in Diffuse Large B-cell Lymphoma Reveals Clinically Relevant Cell Interactions and Recurrent Cellular Neighborhoods
Source: Cancer Immunol Res. 2025 Aug 6;13(10):1674–86. doi: 10.1158/2326-6066.CIR-24-1163 (PMC12485370; doi:10.1158/2326-6066.CIR-24-1163)
Supplement: Figure S12 — Proportions of cells with different RCNs in B2M, HLA-ABC, and HLA-DR positive and negative DLBCLs. [file cir-24-1163_figure_s12_supps12.docx]

**Supplementary Figure 12. Proportions of cells with different RCNs in B2M, HLA-ABC, and HLA-DR positive and negative DLBCLs.**


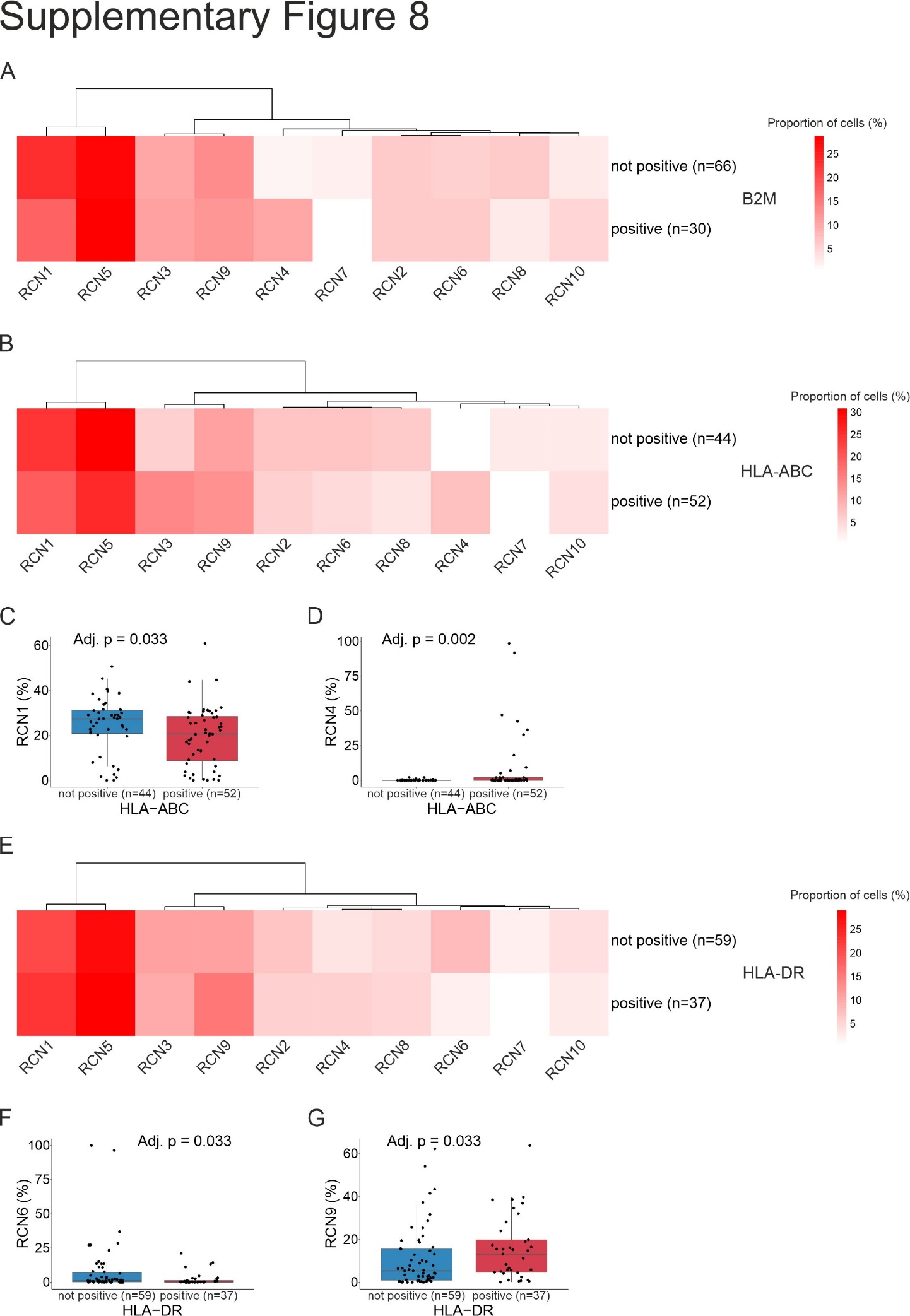


**Supplementary Figure 12. Proportions of cells with different RCNs in B2M, HLA-ABC, and HLA-DR positive and negative DLBCLs.**

A-B) Heatmaps depicting the average proportions depicting the average proportions of cells with B cell rich with immune cells RCN1, CD8^+^ T cell rich RCN2, CD4^+^ T cell rich RCN3, PD-1^+^ cell rich RCN4, immune poor RCN5, M2-like macrophage/non-immune cell rich RCN6, PD-L1^+^ B cell rich RCN7, M2-like macrophage rich RCN8, B-cell rich with T cells RCN9, and PD-L1^+^ M2-like macrophage rich RCN10 neighborhoods in B2M (A) and HLA-ABC (B) positive and negative DLBCLs.

C-D) Boxplots depicting the proportions of cells with RCN1 (C) and RCN4 (D) neighborhoods in HLA-ABC positive and negative DLBCLs.

E) A heatmap depicting the proportions of cells with RCN1-RCN10 neighborhoods in HLA-DR positive and negative DLBCLs.

F-G) Boxplots depicting the proportions of cells with RCN6 (F) and RCN9 (G) neighborhoods in HLA-DR positive and negative DLBCLs.
