# Supplementary material for: Spatial Analysis of the Tumor Microenvironment in Diffuse Large B-cell Lymphoma Reveals Clinically Relevant Cell Interactions and Recurrent Cellular Neighborhoods
Source: Cancer Immunol Res. 2025 Aug 6;13(10):1674–86. doi: 10.1158/2326-6066.CIR-24-1163 (PMC12485370; doi:10.1158/2326-6066.CIR-24-1163)
Supplement: Figure S13 — Clinical impact of the distance between RCNs in DLBCL NOS. [file cir-24-1163_figure_s13_supps13.docx]

**Supplementary Figure 13. Clinical impact of the distance between RCNs in DLBCL NOS.**


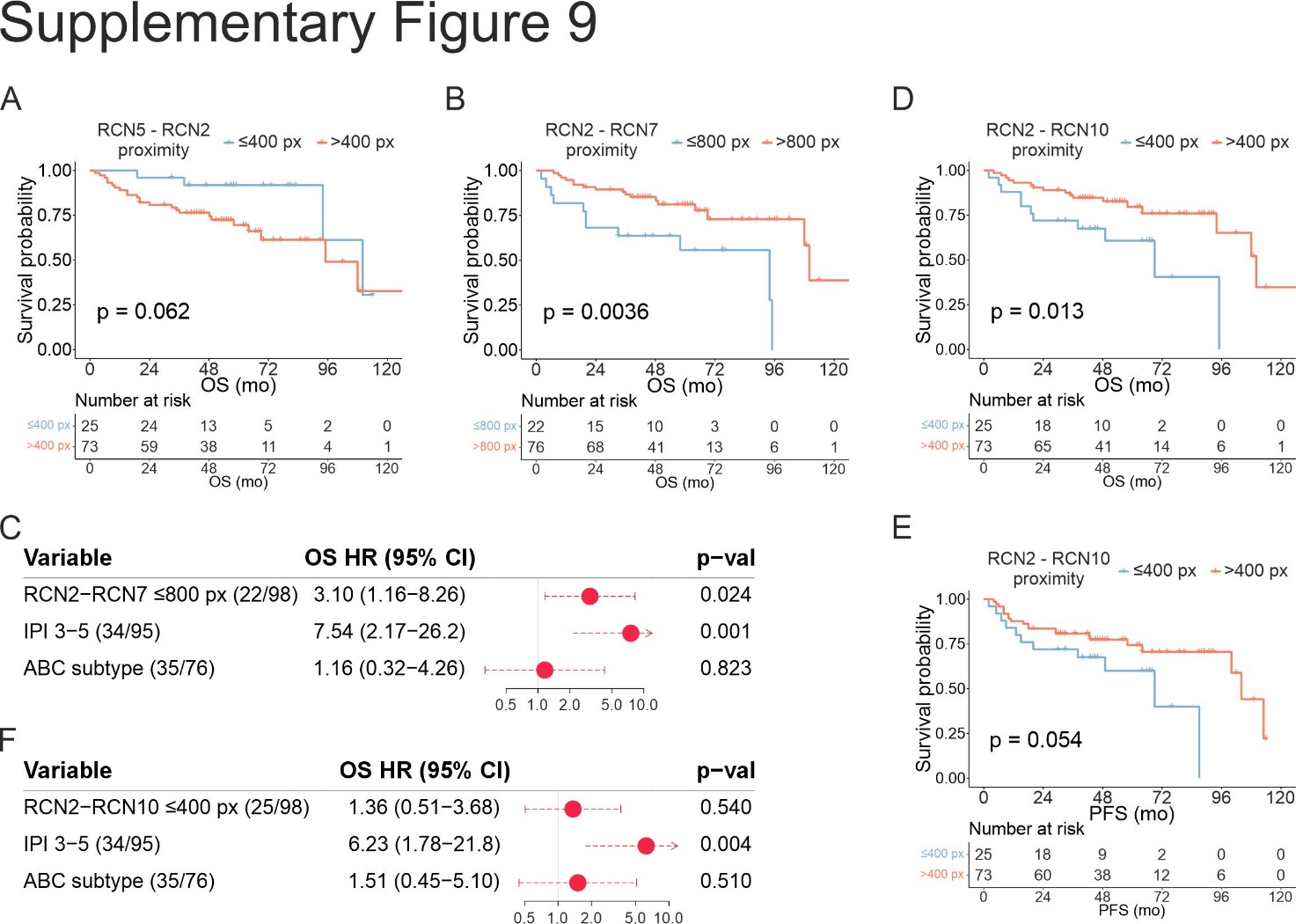


**Supplementary Figure 13. Clinical impact of the distance between RCNs in DLBCL NOS.**

A) Kaplan-Meier (log-rank test) survival plot depicts OS in R-CHOP treated DLBCL NOS patients in whose lymphomas CD8^+^ T cell rich RCN2 neighborhoods are situated very close to immune poor RCN5 neighborhoods and in patients in whose lymphomas these RCNs are situated far from each other or are not present.

B) Kaplan-Meier (log-rank test) survival plot depicts OS in R-CHOP treated DLBCL NOS patients in whose lymphomas PD-L1^+^ B cell rich RCN7 neighborhoods are situated very close or close to CD8^+^ T cell rich RCN2 neighborhoods and in patients in whose lymphomas these RCNs are situated far from each other or are not present.

C) A forest plot visualizing the impact of the proximity of PD-L1^+^ B cell rich RCN7 neighborhoods to CD8^+^ T cell rich RCN2 neighborhoods on OS in a Cox multivariable regression analysis with IPI and COO in R-CHOP treated DLBCL NOS patients.

D-E) Kaplan-Meier (log-rank test) survival plots depict OS (D) and PFS (E) in R-CHOP treated DLBCL NOS patients in whose lymphomas PD-L1^+^ M2-like macrophage rich RCN10 neighborhoods are situated very close to CD8^+^ T cell rich RCN2 neighborhoods and in patients in whose lymphomas these RCNs are situated far from each other or not present.

F) A forest-plot visualizing the impact of the proximity of PD-L1^+^ M2-like macrophage rich RCN10 neighborhoods to CD8^+^ T cell rich RCN2 neighborhoods on OS in a Cox multivariable regression analysis with IPI and COO in R-CHOP treated DLBCL NOS patients.
