# Supplementary material for: Spatial Analysis of the Tumor Microenvironment in Diffuse Large B-cell Lymphoma Reveals Clinically Relevant Cell Interactions and Recurrent Cellular Neighborhoods
Source: Cancer Immunol Res. 2025 Aug 6;13(10):1674–86. doi: 10.1158/2326-6066.CIR-24-1163 (PMC12485370; doi:10.1158/2326-6066.CIR-24-1163)
Supplement: Figure S14 — Proportion of RCNs in lymphomas where these RCNs are situated close and far from each other. [file cir-24-1163_figure_s14_supps14.docx]

**Supplementary Figure 14. Proportion of RCNs in lymphomas where these RCNs are situated close and far from each other.**

**
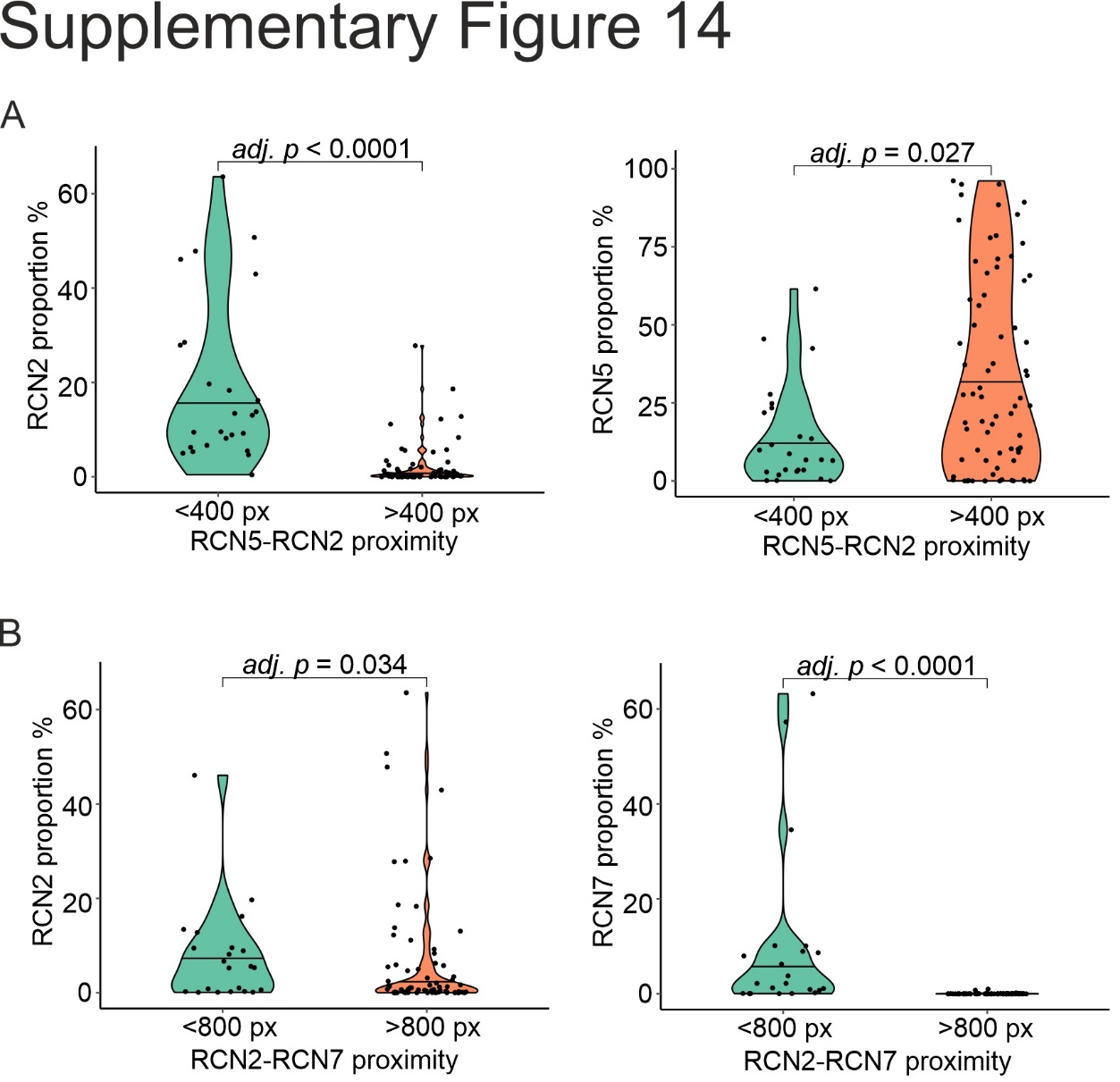
**

**Supplementary Figure 14. Proportion of RCNs in lymphomas where these RCNs are situated close and far from each other.**

A) Violin plots depicting the proportion of CD8^+^ T cell rich RCN2 and immune poor RCN5 in lymphomas where these RCNs are situated close as well as far from each other.

B) Violin plots depicting the proportion of CD8^+^ T cell rich RCN2 and PD-L1^+^ B cell rich RCN7 in lymphomas where these RCNs are situated close as well as far from each other.
