# Supplementary material for: Spatial Analysis of the Tumor Microenvironment in Diffuse Large B-cell Lymphoma Reveals Clinically Relevant Cell Interactions and Recurrent Cellular Neighborhoods
Source: Cancer Immunol Res. 2025 Aug 6;13(10):1674–86. doi: 10.1158/2326-6066.CIR-24-1163 (PMC12485370; doi:10.1158/2326-6066.CIR-24-1163)
Supplement: Figure S15 — Proportions of cells with different RCNs in DLBCLs with different lymphoma ecotypes (LE). [file cir-24-1163_figure_s15_supps15.docx]

**Supplementary Figure 15. Proportions of cells with different RCNs in DLBCLs with different lymphoma ecotypes (LE).**


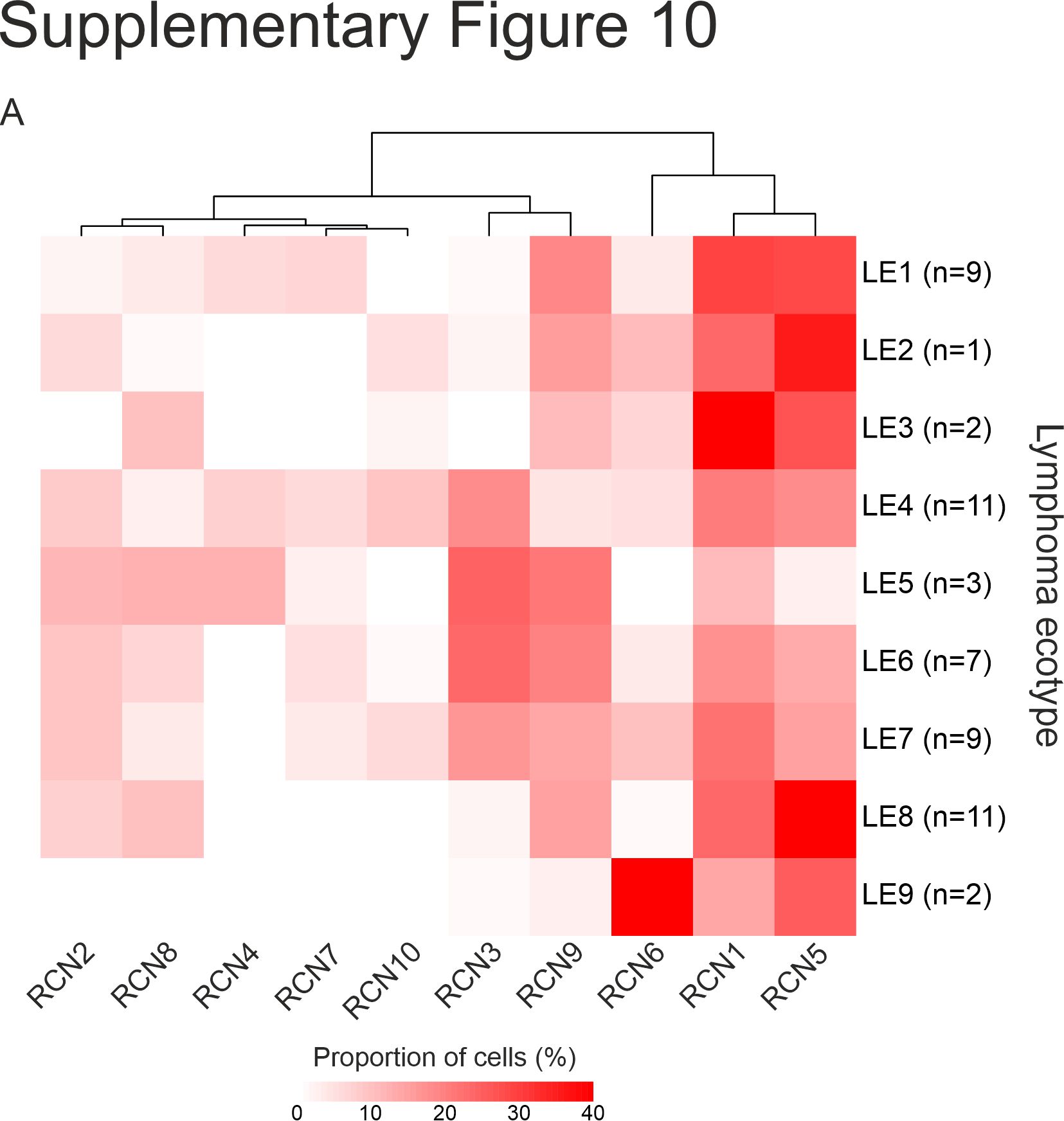


**Supplementary Figure 15. Proportions of cells with different RCNs in DLBCLs with different lymphoma ecotypes (LE).**

A) A heatmap depicting the average proportions of cells with B cell rich with immune cells RCN1, CD8^+^ T cell rich RCN2, CD4^+^ T cell rich RCN3, PD-1^+^ cell rich RCN4, immune poor RCN5, M2-like macrophage/non-immune cell rich RCN6, PD-L1^+^ B cell rich RCN7, M1-like macrophage rich RCN8, B-cell rich with T cells RCN9, and PD-L1^+^ M2-like macrophage rich RCN10 neighborhoods in DLBCLs with LE1-LE9.
